# Chemical Profiling and Redox-Target Mapping of Antioxidant Fractions from *Sphagneticola trilobata*: An Integrated UPLC–ESI–MS/MS, Network Pharmacology, and Molecular Docking Study

**DOI:** 10.3390/ph19081252

**Published:** 2026-08-09

**Authors:** Esraa A. Taema, Wafaa H. B. Hassan, Eman Fikry, May Ahmed El-Sayed, Asmaa M. Arafa, Shaza M. Al-Massarani, Wael M. Abdelmageed, Omar A. Basudan, Afaf E. Abdel Ghani

**Affiliations:** 1 Department of Pharmacognosy, Faculty of Pharmacy, Zagazig University, Zagazig 44519, Egypt; eateema@zu.edu.eg (E.A.T.); wafaahbh@zu.edu.eg (W.H.B.H.); mayalsayed@zu.edu.eg (M.A.E.-S.); asmaaarafa@zu.edu.eg (A.M.A.); aeabdelghani@zu.edu.eg (A.E.A.G.); 2 Department of Pharmacognosy, College of Pharmacy, King Saud University, P.O. Box 2457, Riyadh 11451, Saudi Arabia; salmassarani@ksu.edu.sa; 3 Department of Biochemistry, Molecular Biology, and Biophysics, University of Minnesota, Saint Paul, MN 55108, USA; mosta054@umn.edu

**Keywords:** *Sphagneticola trilobata*, *Wedelia trilobata*, antioxidant activity, oxidative stress, UPLC–ESI–MS/MS, flavanone, chalcone, ADME, in silico analysis

## Abstract

**Background/Objectives**: *Sphagneticola trilobata* is a phytochemically rich medicinal plant, yet its antioxidant-active fractions and redox-related mechanisms remain incompletely characterized. This study combined metabolite profiling, antioxidant screening, network pharmacology, pharmacokineticprediction, and molecular docking to characterize active flower and leaf fractions and prioritize antioxidant-related targets. **Methods**: Petroleum ether, methylene chloride, and ethyl acetate fractions of *S. trilobata* flowers and leaves were analyzed by ultra-performance liquid chromatography coupled with electrospray ionization tandem mass spectrometry (UPLC–ESI–MS/MS). The predominant metabolite was isolated and spectroscopically characterized. Antioxidant activity was assessed using 2,2′-azinobis(3-ethylbenzothiazoline-6-sulphonic acid) (ABTS) and ferric reducing antioxidant power (FRAP). Metabolites from the most active fractions were investigated by target prediction, protein–protein interaction (PPI) analysis, enrichment analysis, SwissADME, and docking. **Results**: UPLC–ESI–MS/MS tentatively identified 59, 18, and 16 metabolites in the petroleum ether, methylene chloride, and ethyl acetate fractions, respectively, including diterpenes, phenolic acids, flavonoids, fatty acids, triterpenes, and coumarin-related metabolites. Butein was isolated as the predominant metabolite from the ethyl acetate flower fraction, which showed the strongest antioxidant activity, with IC_50_ values of 5.85 ± 0.14 µg/mL in ABTS and 9.11 ± 0.75 µg/mL in FRAP, followed by the ethyl acetate leaf fraction. Network analysis identified 61 and 82 antioxidant-relevant targets for the flower and leaf ethyl acetate fractions, respectively, with enrichment in inflammation-, apoptosis-, transcription-, hypoxia-, lipid stress-, and kinase-related pathways. Docking suggested fraction-specific compatibility with EGFR/PTGS2/STAT3 for flower metabolites and AKT1/PTGS2 for leaf metabolites. **Conclusions**: *S. trilobata* ethyl acetate fractions, particularly the flower fraction, represent promising antioxidant sources with experimentally supported activity and computationally prioritized redox-related hypotheses requiring further validation.

## 1. Introduction

*Sphagneticola trilobata* (L.) Pruski, synonym *Wedelia trilobata* (L.) Hitchc., belongs to Asteraceae, one of the largest families of flowering plants. It is a perennial invasive species widely distributed in tropical and subtropical regions, where it can damage farmlands and suppress the growth of neighboring plants [1]. Despite its ecological impact, *W. trilobata* (*S. trilobata*) has been used in traditional medicine for the management of inflammatory conditions [2]. It has also been reported for the treatment of muscle pain, rheumatism, sores, swelling, and arthritis-related pain [3,4,5]. Phytochemical investigations have shown that this species contains diverse secondary metabolites, including ent-kaurane diterpenes, sesquiterpene lactones, triterpenes, flavonoids, polyacetylenes, and steroids [6]. Among the ent-kaurane diterpenoids reported from the species are kaurenoic acid, grandiflorenic acid, and wedelobatins A and B [3,7]. Reported eudesmanolide sesquiterpene lactones include wedelolides G and H and trilobolide-6-*O*-isobutyrate [8], whereas 5,4′-dihydroxy-7-methoxyflavone and 5,3′,4′-trihydroxy-7-methoxyflavone are among the flavonoids isolated from the species [8]. Triterpenoid constituents isolated from the flowers include friedelan-3*β*-ol, erythrodiol, and oleanane derivatives, while the reported sterols include gramisterol, stigmasterol, and *β*-sitosterol 3-*O*-*β*-D-glucopyranoside [9].

*S. trilobata* has attracted increasing research interest as a phytochemically rich medicinal plant with reported biological activities, while studies on its Egyptian material remain relatively limited [10,11,12]. Recently, a comparative metabolomics study expanded the phytochemical understanding of *S. trilobata* by demonstrating organ-dependent metabolic variation between flowerheads and leaves and associating the stronger antioxidant activity of flowerheads with their enrichment in flavonoids and phenolic acids [13]. However, fraction-level profiling of antioxidant-active fractions, isolation of predominant metabolites, and redox-target mapping through network pharmacology, ADME prediction, and molecular docking remain insufficiently explored.

Oxidative stress results from an imbalance between reactive oxygen species (ROS) generation and endogenous antioxidant defense systems. It is closely associated with the pathophysiology of several disorders, including cancer, atherosclerosis, cardiovascular disease, hypertension, rheumatoid arthritis, neurodegenerative diseases, and AIDS [14]. Excessive ROS can damage cellular macromolecules, including proteins, lipids, and DNA, leading to impaired cellular function and disease progression. Although endogenous antioxidant enzymes, such as superoxide dismutase, glutathione reductase, glutathione peroxidase, and catalase, contribute to cellular defense, these systems may be insufficient under sustained oxidative stress [14]. Therefore, natural antioxidants derived from plant sources remain of considerable interest as potential agents for reducing oxidative damage and supporting cellular redox balance. This interest has been further encouraged by concerns regarding the safety of some synthetic antioxidants, including butylated hydroxyanisole and butylated hydroxytoluene [14].

Beyond conventional antioxidant assays, which primarily assess radical-scavenging and reducing capacities, network pharmacology can provide an exploratory framework for linking plant secondary metabolites to putative molecular targets and pathways potentially associated with antioxidant activity. In parallel, ADME prediction and molecular docking may help prioritize metabolites with favorable predicted pharmacokinetic profiles and assess the structural plausibility of selected metabolite–target interactions, thereby informing mechanistic hypotheses for bioactive natural product fractions [15].

Accordingly, the present study aimed to profile *S. trilobata* flower and leaf fractions of different polarities using UPLC–ESI–MS/MS, isolate and characterize the predominant metabolite, and evaluate the antioxidant activity of the fractions and isolated compound using ABTS and FRAP assays. The metabolite profiles of the most active fractions were further integrated with network pharmacology, ADME prediction, and molecular docking to prioritize putative targets and pathways potentially associated with antioxidant activity, assess the predicted pharmacokinetic properties of the identified metabolites, and examine the structural plausibility of selected metabolite–target interactions.

## 2. Results

### 2.1. UPLC–ESI–MS/MS Chemical Profiling of S. trilobata Fractions

UPLC–ESI–MS/MS analysis of the petroleum ether, methylene chloride, and ethyl acetate fractions of *S. trilobata* flowers and leaves resulted in tentative metabolite annotations in positive and negative ionization modes. These comprised 59 metabolites in the petroleum ether fractions, 18 in the methylene chloride fractions, and 16 unique tentative metabolite annotations across STF-EAF and STL-EAF. Unless otherwise indicated, these LC–MS/MS assignments represent tentative candidate structures. Diterpene-related peaks showing differences in retention time and/or product-ion profiles were treated as distinct chromatographic features; however, their exact positional and stereochemical identities were not considered definitive in the absence of authentic reference standards.

The petroleum ether fractions showed the broadest chemical diversity and were mainly enriched in lipophilic constituents, including ent-kaurane diterpenes, fatty acids and their derivatives, triterpenes, sterols, coumarins, and related metabolites. Twelve metabolites were shared between flowers and leaves, whereas 25 were flower-specific and 22 were leaf-specific. The tentative assignment of diterpenoid-related metabolites was supported by characteristic neutral losses of H_2_O, COOH, CH_2_OH, and acyl substituents, while fatty acid derivatives showed typical alkyl-chain and ester-associated fragments. These annotations were supported by comparison with reported data for related diterpenes, triterpenes, fatty acid derivatives, phenolic acids, sterols, and coumarins [1,3,6,7,9,16,17,18,19,20,21,22,23,24,25,26,27,28,29,30,31,32,33,34,35,36,37,38,39,40,41,42,43]. In addition, the previous reporting of several diterpenes in *W. trilobata* provided further phytochemical support for the proposed annotations [3,7,21,29]. The complete annotation of the petroleum ether fractions is provided in Supplementary Table S1, while the corresponding chromatograms, selected structures, and representative MS/MS spectra are shown in Supplementary Figures S1–S3, respectively.

The methylene chloride fractions contained 18 non-redundant tentatively identified metabolites listed in Supplementary Table S2. Of these, nine metabolites were detected in the flower fraction and ten in the leaf fraction. Butin was the only metabolite shared between the two organs, corresponding to eight flower-specific and nine leaf-specific metabolites. The identified metabolites included flavonoids and flavonoid-related compounds, phenolic acids, diterpenes, fatty acids, one alcohol, one coumarin, and one glycoside. Representative flower-associated metabolites included vanillic acid, butin, butein, isoferulic acid, liquiritigenin, luteolin, diosmetin, kaempferol, and hesperetin, whereas the leaf fraction contained butin, ferulic acid, diterpenoid-related metabolites, fatty acids, hexaethylene glycol, naueloside G, and coumarin. The tentative assignments were supported by diagnostic molecular ions and MS/MS fragments characteristic of phenolic acids, flavonoids, and diterpenoid-related metabolites, including losses of OCH_3_, CO_2_, glycosidic residues, and flavonoid ring-cleavage ions. These annotations were supported by comparison with reported MS/MS and phytochemical data for related flavonoids, phenolic acids, diterpenes, fatty acids, coumarins, and glycosidic metabolites [6,8,16,24,26,44,45,46,47,48,49,50,51,52,53,54,55,56,57,58]. The corresponding chromatograms, selected structures, and representative MS/MS spectra are presented in Supplementary Figures S4 and S5.

**Figure 1:**
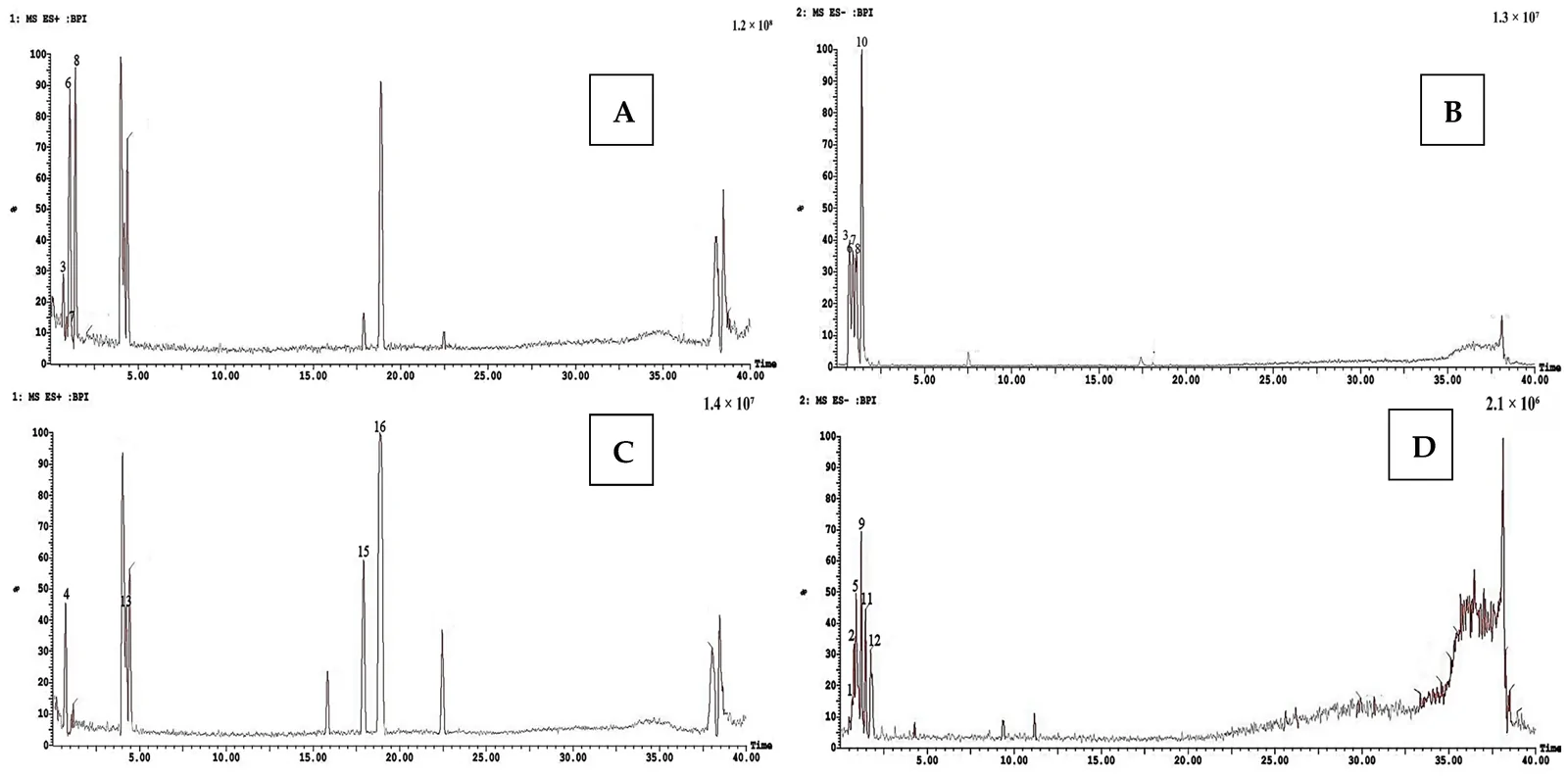
UPLC–ESI–MS/MS chromatograms of the ethyl acetate fractions of *S. trilobata* flowers and leaves in positive ionization mode (**A**,**C**) and negative ionization mode (**B**,**D**), respectively.

**Figure 2:**
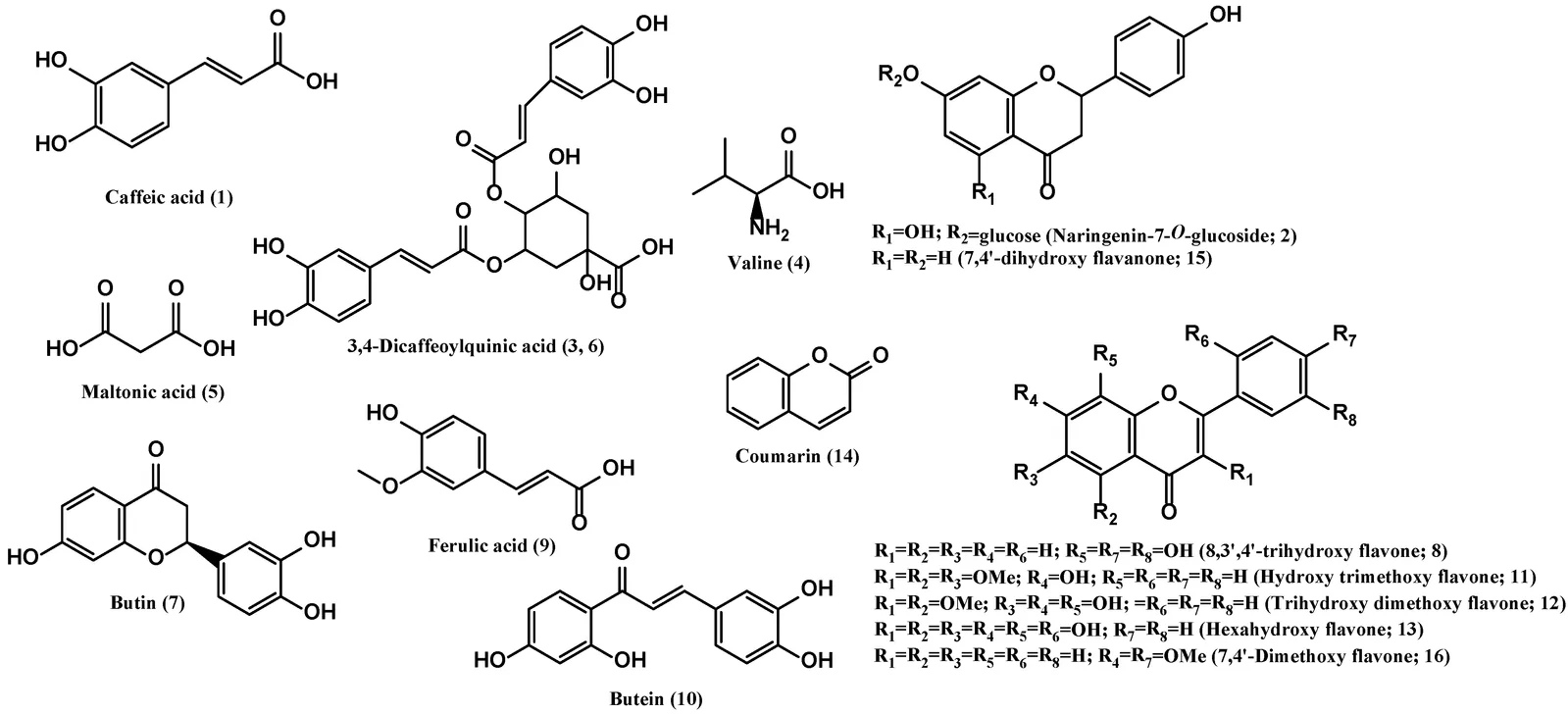
Chemical structures of all metabolites tentatively identified in the ethyl acetate fractions of *S. trilobata* flowers and leaves by UPLC–ESI–MS/MS. Compound numbers correspond to those listed in Table 1.

The ethyl acetate fractions displayed a more polar phenolic/flavonoid-rich profile and were selected for antioxidant-guided in silico analyses. Across STF-EAF and STL-EAF, 16 unique tentative metabolite annotations were recorded (Table 1). STF-EAF contained five metabolites, namely 3,4-dicaffeoylquinic acid (3), dicaffeoylquinic acid isomer (6), butin (7), 8,3′,4′-trihydroxyflavone (8), and butein (10). STL-EAF contained twelve metabolites, namely caffeic acid (1), naringenin-7-*O*-glucoside (2), valine (4), maltonic acid (5), butin (7), ferulic acid (9), 4′-hydroxy-5,6,7-trimethoxyflavone (11), 3,5,8-trihydroxy-6,7-dimethoxyflavone (12), 3,3′,4′,5,5′,7-hexahydroxyflavone (13), coumarin (14), 7,4′-dihydroxyflavanone (15), and 7,4′-dimethoxyflavone (16). Butin (7) was the only metabolite shared between STF-EAF and STL-EAF, whereas compounds 3, 6, 8, and 10 were specific to STF-EAF, and compounds 1, 2, 4, 5, 9, and 11–16 were specific to STL-EAF. Their tentative identification was supported by protonated or deprotonated molecular ions and diagnostic MS/MS fragments related to losses of glycosidic residues, caffeoyl/quinic acid moieties, OCH_3_, CO_2_, and characteristic flavonoid ring-cleavage ions. The ethyl acetate metabolites, including retention times, molecular ions, diagnostic fragments, relative abundance, and references, are listed in Table 1. The corresponding UPLC–ESI–MS/MS chromatograms are shown in Figure 1. The chemical structures of all ethyl acetate metabolites are shown in Figure 2, while representative MS/MS spectra are provided in Supplementary Figure S6.

### 2.2. Characterization of the Isolated Compound

UPLC–ESI–MS/MS profiling indicated that butein was the dominant detected metabolite in the flower ethyl acetate fraction based on relative abundance. Accordingly, chromatographic purification of this fraction afforded butein as the major isolated compound, whose structure was established using UV, ESI-MS, ^1^H-NMR, ^13^C-NMR, HSQC, and HMBC analyses.

**Figure 3:**
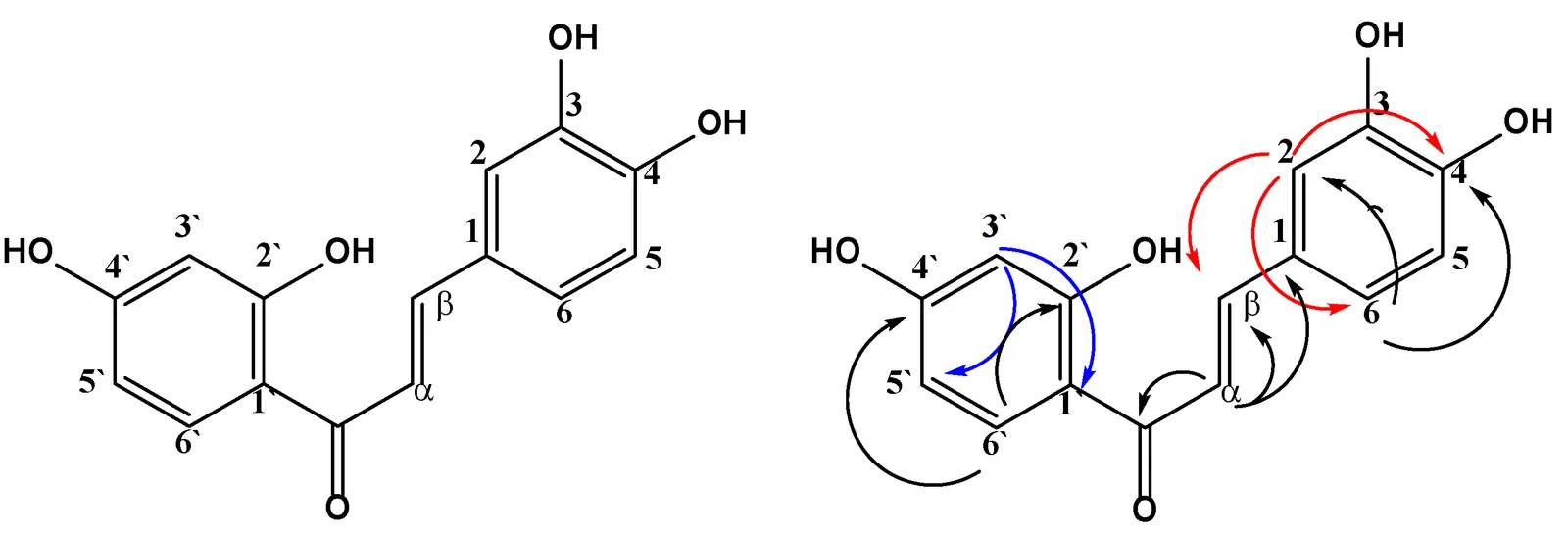
Chemical structure of butein and its key HMBC correlations.

Butein (Figure 3) was obtained as orange-yellow needles (150 mg), with an R_f_ value of 0.58 using benzene: ethyl acetate: acetic acid (5:3.5:0.5). UV λ_max_ (MeOH) nm: 380 (sh), 310 (sh), 277; +NaOMe: 440 (sh), 310 (sh); +AlCl_3_: 438 (sh), 277; +AlCl_3_/HCl: 438 (sh), 380 (sh), 277; +NaOAc: 410 (sh), 340; +NaOAc/H_3_BO_3_: 420 (sh), 277. ^1^H-NMR (400 MHz, CD_3_OD) δ ppm: 6.19 (1H, d, *J* = 2.4 Hz, H-3′), 6.32 (1H, dd, *J* = 8.8, 2.4 Hz, H-5′), 6.73 (1H, d, *J* = 8.0 Hz, H-5), 7.07 (1H, dd, *J* = 8.4, 2.0 Hz, H-6), 7.09 (1H, d, *J* = 2.0 Hz, H-2), 7.46 (1H, d, *J* = 15.2 Hz, H-*α*), 7.64 (1H, d, *J* = 15.2 Hz, H-*β*), 7.86 (1H, d, *J* = 8.8 Hz, H-6′). ^13^C-NMR (100 MHz, CD_3_OD) δ ppm: 102.4 (C-3′), 107.7 (C-5′), 113.3 (C-2), 114.3 (C-1′), 115.2 (C-5), 116.9 (C-α), 122.2 (C-6), 127.0 (C-1), 131.8 (C-6′), 144.7 (C-β), 145.5 (C-3), 148.6 (C-4), 165.1 (C-2′), 166.1 (C-4′), 192.1 (C=O). Negative-ion ESI-MS showed ion peaks at *m*/*z* 271 [M−H]^−^, 543 [2M−H]^−^, 136, and 135. Based on these spectral data and comparison with the literature [67,68], the compound was identified as butein.

### 2.3. Antioxidant Activity

**Figure 4:**
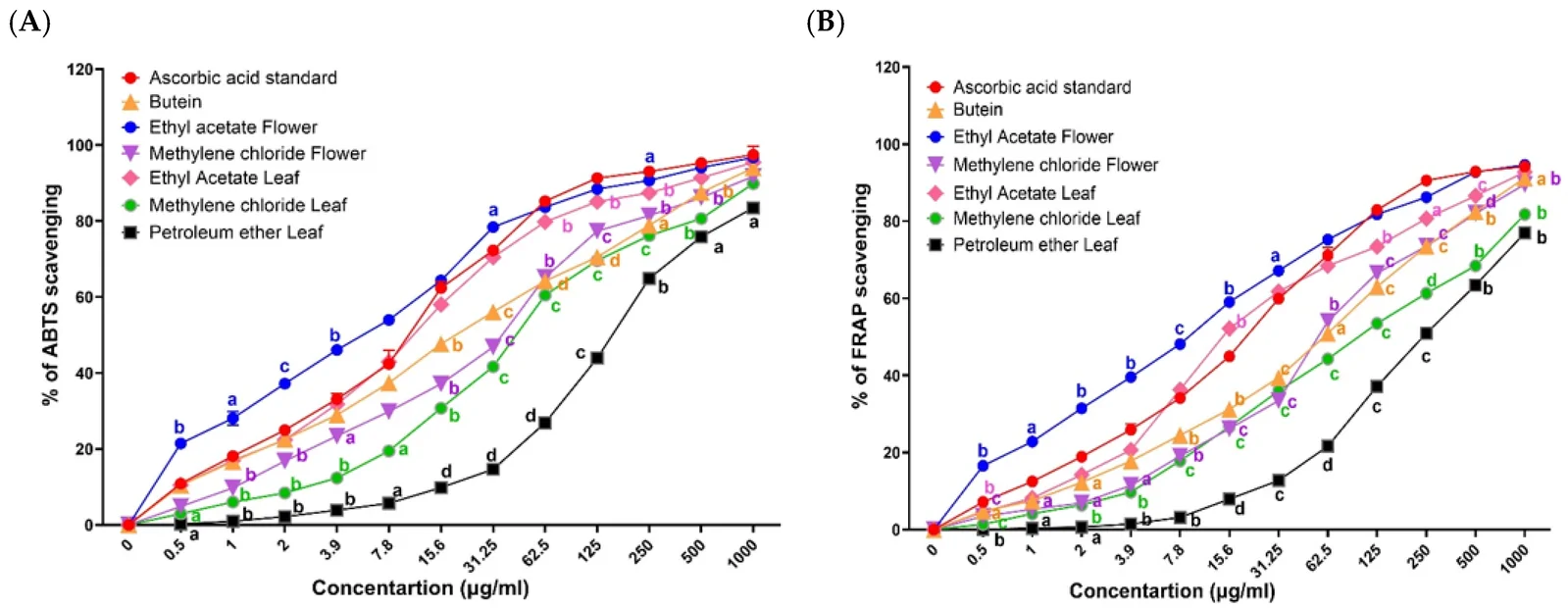
Antioxidant activity of *S. trilobata* fractions, isolated butein, and ascorbic acid. (**A**) ABTS radical scavenging activity. (**B**) Ferric reducing antioxidant power. Data are expressed as mean ± SD of three determinations (*n* = 3). Statistical significance was determined using two-way ANOVA followed by Bonferroni’s multiple comparisons test. Superscript letters a–d indicate significant differences versus ascorbic acid at *p* < 0.05, *p* < 0.01, *p* < 0.001, and *p* < 0.0001, respectively.

The antioxidant activity of the tested samples was evaluated using ABTS radical scavenging and ferric reducing antioxidant power (FRAP) assays over a concentration range of 0–1000 µg/mL. The tested samples included the methylene chloride and ethyl acetate flower fractions, the petroleum ether, methylene chloride, and ethyl acetate leaf fractions, and isolated butein, using ascorbic acid as the reference antioxidant. The calculated IC_50_ values are summarized in Table 2, while the concentration-response profiles are presented in Figure 4. The corresponding IC_50_ bar plots are provided as Supplementary Figure S7. Lower IC_50_ values indicate stronger antioxidant activity.

In the ABTS assay, all tested samples showed concentration-dependent radical scavenging activity. Among the tested fractions, the ethyl acetate flower fraction exhibited the strongest ABTS radical scavenging activity, with an IC_50_ value of 5.85 ± 0.14 µg/mL. This activity was higher than that of ascorbic acid, which showed an IC_50_ value of 10.66 ± 0.63 µg/mL. The ethyl acetate leaf fraction also showed pronounced activity, with an IC_50_ value of 11.45 ± 0.63 µg/mL, followed by isolated butein (20.00 ± 1.59 µg/mL), the methylene chloride flower fraction (37.45 ± 1.37 µg/mL), and the methylene chloride leaf fraction (45.10 ± 1.84 µg/mL). The petroleum ether leaf fraction was the least active sample in this assay, with an IC_50_ value of 161.35 ± 4.92 µg/mL. At the highest tested concentration, the ethyl acetate flower and leaf fractions showed strong ABTS radical scavenging responses, reaching 96.73% and 95.48%, respectively.

A similar trend was observed in the FRAP assay, where the ethyl acetate flower fraction again displayed the strongest reducing capacity, with an IC_50_ value of 9.11 ± 0.75 µg/mL. The ethyl acetate leaf fraction ranked second among the tested fractions, with an IC_50_ value of 14.53 ± 0.92 µg/mL, followed by ascorbic acid (20.89 ± 1.25 µg/mL), the methylene chloride flower fraction (56.60 ± 1.82 µg/mL), butein (59.96 ± 1.25 µg/mL), the methylene chloride leaf fraction (101.41 ± 3.47 µg/mL), and the petroleum ether leaf fraction (241.29 ± 6.09 µg/mL). At 1000 µg/mL, the ethyl acetate flower fraction showed the highest FRAP response, reaching 94.61 ± 0.75%, while the ethyl acetate leaf fraction, ascorbic acid, butein, methylene chloride flower fraction, methylene chloride leaf fraction, and petroleum ether leaf fraction showed responses of 92.73 ± 0.29%, 94.19 ± 0.40%, 91.20 ± 0.56%, 89.56 ± 0.17%, 81.79 ± 0.87%, and 76.93 ± 1.09%, respectively.

Based on their superior antioxidant activity in both ABTS and FRAP assays, the ethyl acetate flower and leaf fractions were selected for subsequent network pharmacology, ADME prediction, and molecular docking analyses.

### 2.4. Network Pharmacology Analysis of the Active Ethyl Acetate Fractions

#### 2.4.1. Predicted Target Profiling and Antioxidant-Relevant Target Intersection

After LC–MS/MS data curation, four non-redundant metabolites from the *S. trilobata* flower ethyl acetate fraction (STF-EAF) and twelve from the *S. trilobata* leaf ethyl acetate fraction (STL-EAF) were subjected to SwissTargetPrediction. For STF-EAF, approximately 400 raw compound-target entries were reduced to 233 unique predicted targets after official gene symbol standardization and duplicate removal (Supplementary Table S3). For STL-EAF, 1263 raw compound-target entries were reduced to 448 unique predicted targets using the same procedure (Supplementary Table S4).

**Figure 5:**
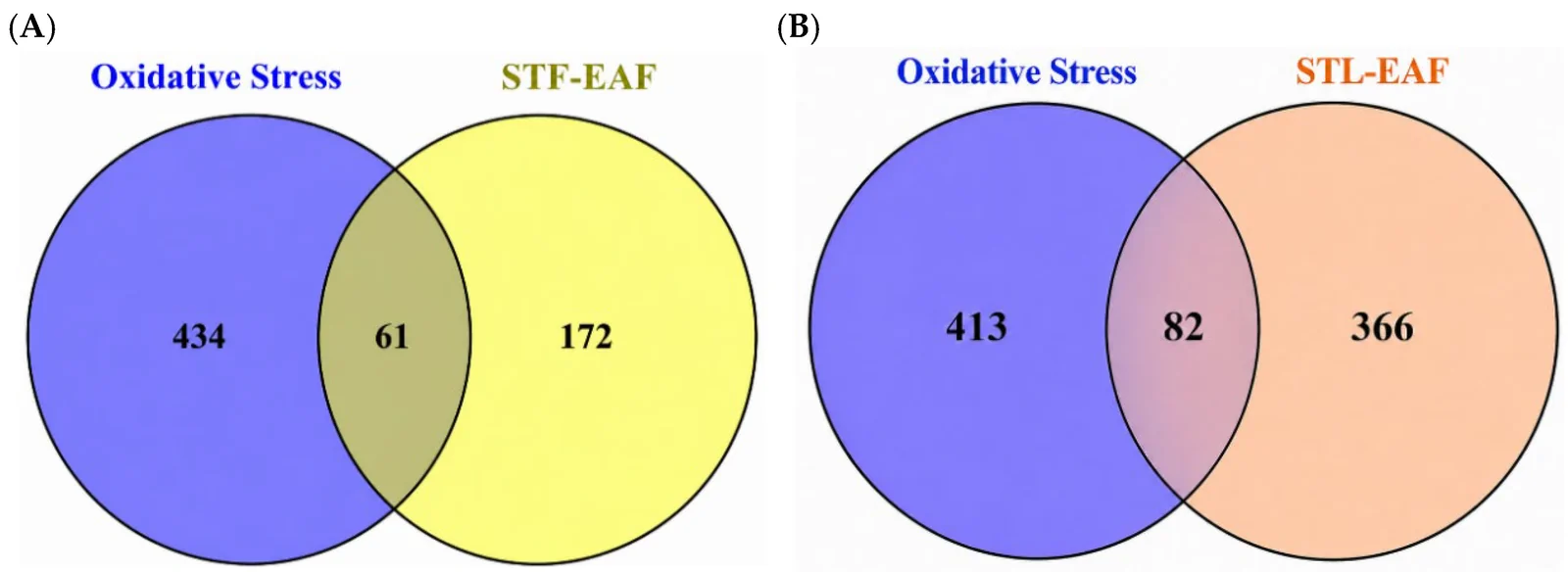
Venn diagrams showing the intersection between oxidative stress-related genes and the predicted targets of (**A**) the *S. trilobata* flower ethyl acetate fraction (STF-EAF) and (**B**) the *S. trilobata* leaf ethyl acetate fraction (STL-EAF).

Oxidative stress-related genes retrieved from GeneCards and OMIM were merged and deduplicated, yielding 495 unique genes (Supplementary Table S5). Intersecting this gene set with the fraction-specific predicted targets identified 61 overlapping targets for STF-EAF and 82 for STL-EAF (Figure 5 and Supplementary Tables S6 and S7). These overlapping targets were retained as putative antioxidant-relevant targets for subsequent PPI network construction, compound-target network analysis, and functional enrichment.

#### 2.4.2. PPI Network Construction and Hub Target Identification

**Figure 6:**
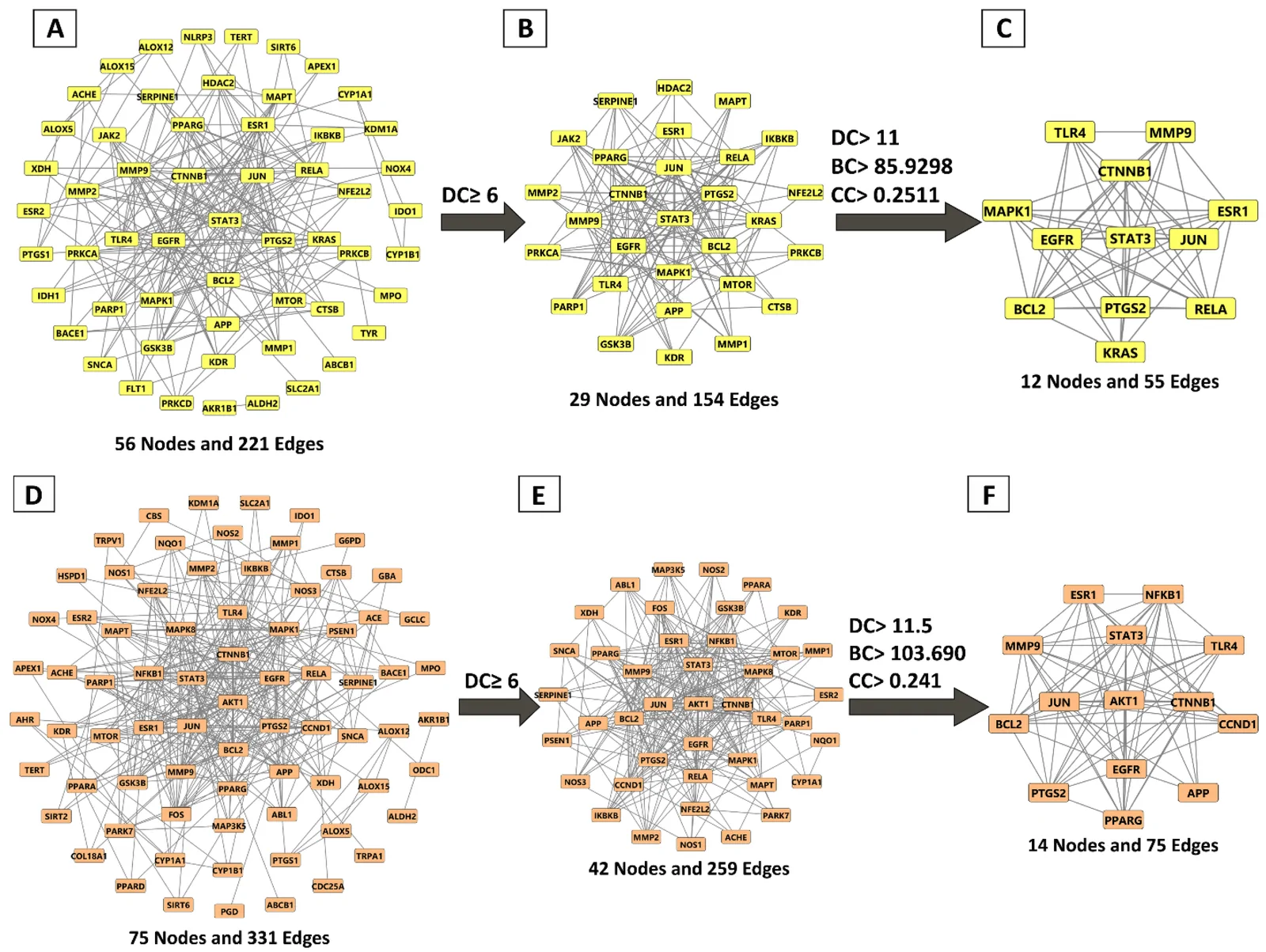
PPI network construction and topological screening of STF-EAF and STL-EAF antioxidant-related targets. (**A**) Initial STF-EAF PPI network after removal of disconnected nodes. (**B**) STF-EAF primary subnetwork after degree-based filtering. (**C**) STF-EAF core hub network after combined DC, BC, and CC filtering. (**D**) Initial STL-EAF PPI network after removal of disconnected nodes. (**E**) STL-EAF primary subnetwork after degree-based filtering. (**F**) STL-EAF core hub network after combined DC, BC, and CC filtering. STF-EAF, *S. trilobata* flower ethyl acetate fraction; STL-EAF, *S. trilobata* leaf ethyl acetate fraction; DC, degree centrality; BC, betweenness centrality; CC, closeness centrality.

The antioxidant-relevant overlapping targets of STF-EAF and STL-EAF were separately submitted to STRING using a high-confidence interaction score of 0.7, with disconnected nodes hidden before downstream analysis. For STF-EAF, the initial PPI network contained 56 nodes and 221 edges (Figure 6A and Supplementary Table S8). Cytoscape/CytoNCA analysis followed by degree-based filtering (DC ≥ 6) generated a primary subnetwork of 29 nodes and 154 edges (Figure 6B and Supplementary Table S9). Further median-based filtering using DC ≥ 11, BC ≥ 85.9298, and CC ≥ 0.2511 produced a core PPI network of 12 nodes and 55 edges (Figure 6C and Table 3).

Using the same workflow, STL-EAF generated an initial PPI network of 75 nodes and 331 edges (Figure 6D and Supplementary Table S10). Degree-based filtering (DC ≥ 6) produced a primary subnetwork of 42 nodes and 259 edges (Figure 6E and Supplementary Table S11). Subsequent filtering using DC ≥ 11.5, BC ≥ 103.690, and CC ≥ 0.241 resulted in a final core PPI network of 14 nodes and 75 edges (Figure 6F and Table 3).

The core targets from both fractions were ranked in descending order according to degree centrality and were used for subsequent functional enrichment and mechanistic interpretation.

#### 2.4.3. Compound-Target Network Analysis

**Figure 7:**
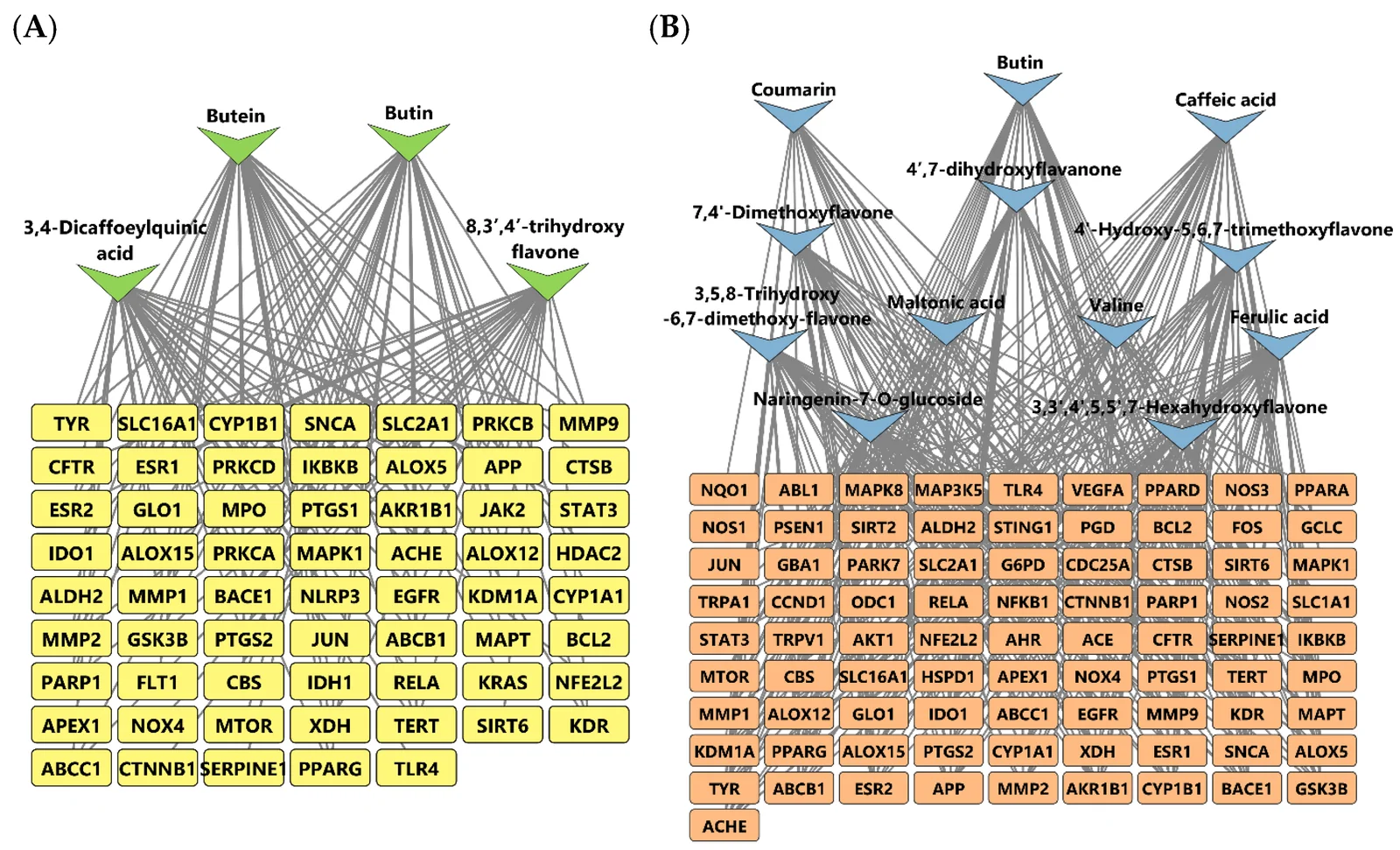
Compound-target networks of (**A**) the *S. trilobata* flower ethyl acetate fraction (STF-EAF) and (**B**) the *S. trilobata* leaf ethyl acetate fraction (STL-EAF). Triangular nodes represent metabolites, rectangular nodes represent antioxidant-relevant target proteins, and edges indicate predicted compound-target associations.

Compound-target networks were constructed to visualize the predicted associations between STF-EAF and STL-EAF metabolites and their antioxidant-relevant overlapping targets (Figure 7). In STF-EAF, the four metabolites showed comparable target coverage, with butein showing the highest degree score (34), followed by 3,4-dicaffeoylquinic acid (33), while butin and 8,3′,4′-trihydroxyflavone each showed a degree score of 30 (Supplementary Table S12).

In STL-EAF, ferulic acid showed the highest degree score (35), followed by 3,5,8-trihydroxy-6,7-dimethoxyflavone and 7,4′-dimethoxyflavone, each with a degree score of 33. Caffeic acid and 4′-hydroxy-5,6,7-trimethoxyflavone also showed high connectivity, with degree scores of 32 each (Supplementary Table S13). Overall, these networks support the multi-compound, multi-target nature of both fractions; however, degree-based ranking reflects predicted target connectivity rather than direct evidence of individual antioxidant activity.

#### 2.4.4. Functional Enrichment Analysis of Core Hub Targets

**Figure 8:**
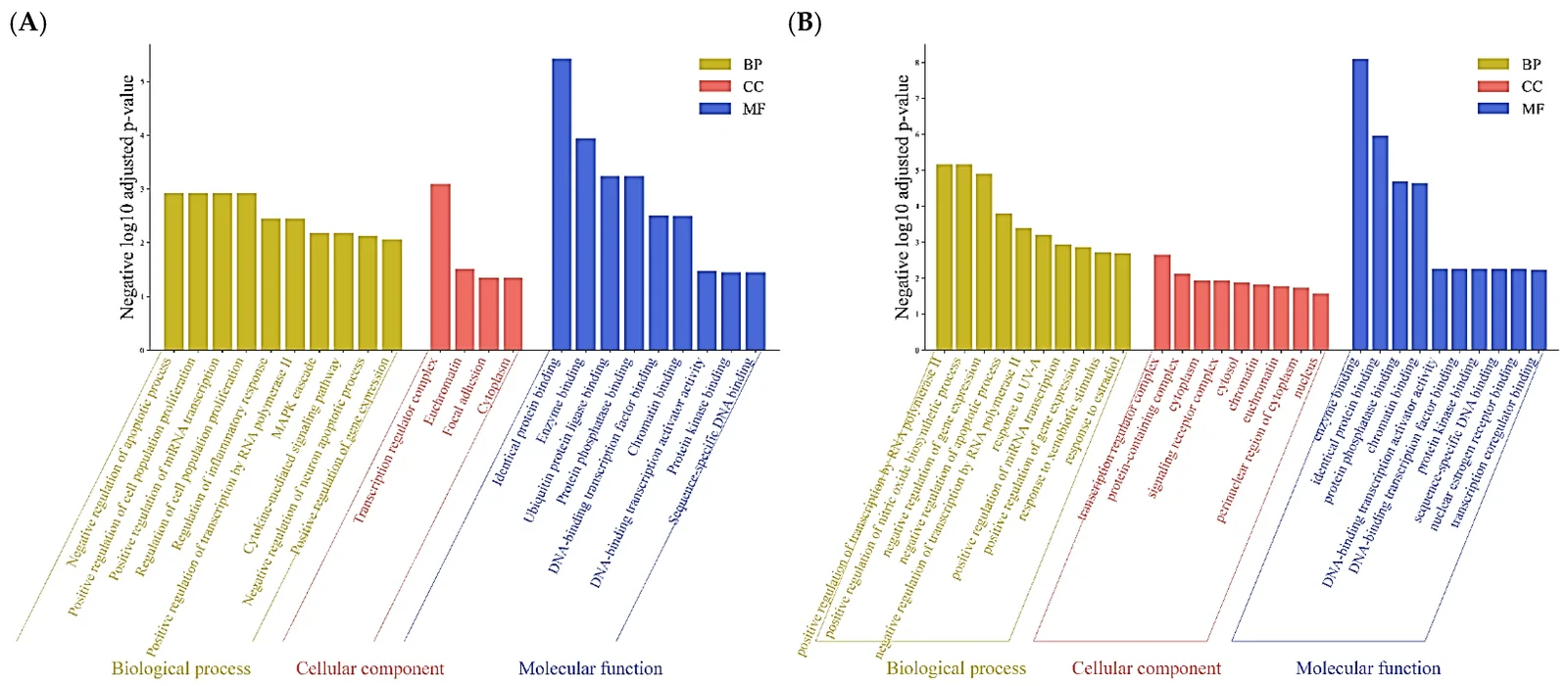
GO enrichment analysis of (**A**) STF-EAF and (**B**) STL-EAF core hub targets. Selected biological process (BP), cellular component (CC), and molecular function (MF) terms are shown. Enriched terms were selected using Benjamini-adjusted *p*-value ≤ 0.05. STF-EAF, *S. trilobata* flower ethyl acetate fraction; STL-EAF, *S. trilobata* leaf ethyl acetate fraction.

**Figure 9:**
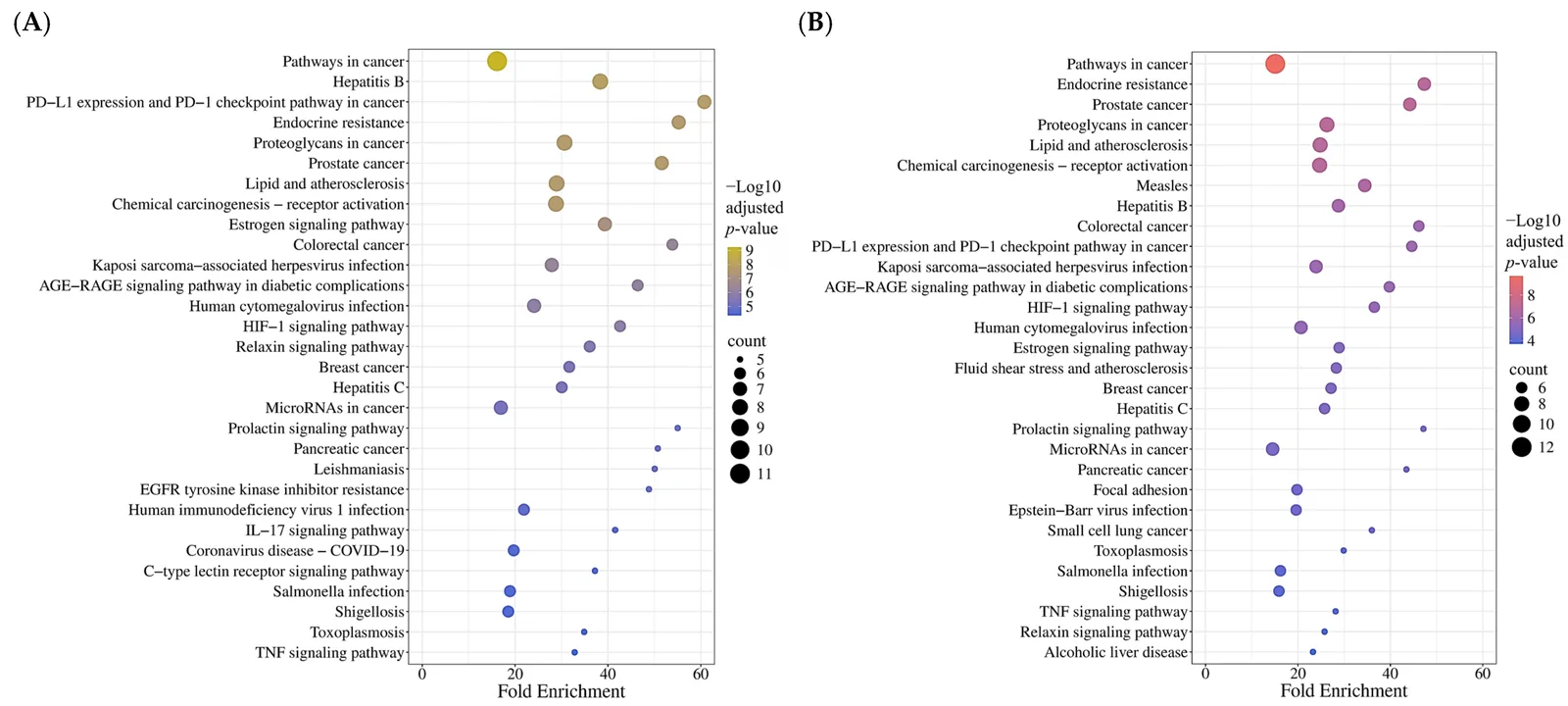
KEGG pathway enrichment analysis of (**A**) STF-EAF and (**B**) STL-EAF core hub targets. Bubble plots show the top 30 enriched KEGG pathways selected using Benjamini-adjusted *p*-value ≤ 0.05. STF-EAF, *S. trilobata* flower ethyl acetate fraction; STL-EAF, *S. trilobata* leaf ethyl acetate fraction.

DAVID-based functional enrichment analysis was performed separately for STF-EAF and STL-EAF core hub targets, using Benjamini-adjusted *p*-value ≤0.05 as the significance threshold. For STF-EAF, the 12 core hub targets yielded 25 BP terms, 4 CC terms, 9 MF terms, and 104 KEGG pathways (Figure 8A and Figure 9A; Supplementary Tables S14–S17). The enriched GO terms were mainly related to apoptosis regulation, inflammatory response, MAPK cascade, cytokine-mediated signaling, transcriptional regulation, focal adhesion, chromatin-related regulation, and kinase/protein-binding functions. KEGG analysis highlighted oxidative stress-relevant pathways, including AGE-RAGE signaling in diabetic complications, HIF-1 signaling, TNF signaling, IL-17 signaling, lipid and atherosclerosis, and C-type lectin receptor signaling.

For STL-EAF, enrichment analysis identified 34 BP terms, 9 CC terms, 20 MF terms, and 89 KEGG pathways (Figure 8B and Figure 9B; Supplementary Tables S18–S21). The enriched GO terms were mainly associated with transcriptional regulation, nitric oxide biosynthesis, apoptosis regulation, miRNA transcription, xenobiotic and UV-A responses, chromatin-associated regulation, signaling receptor complexes, kinase/phosphatase binding, DNA-binding transcription factor activity, and transcription coregulator binding. KEGG enrichment highlighted lipid and atherosclerosis, AGE-RAGE signaling in diabetic complications, HIF-1 signaling, TNF signaling, fluid shear stress and atherosclerosis, focal adhesion, and relaxin signaling.

Overall, both fractions were enriched in redox-sensitive modules related to inflammation, apoptosis, transcriptional control, hypoxia response, lipid-associated stress, and kinase-mediated signaling. STF-EAF showed greater emphasis on MAPK-, cytokine-, IL-17-, and C-type lectin receptor-related pathways, whereas STL-EAF was additionally characterized by nitric oxide biosynthesis, xenobiotic/UV response, fluid shear stress, and relaxin-related signaling.

### 2.5. Predicted Pharmacokinetic and Drug-likeness Properties of STF-EAF and STL-EAF Metabolites

The ADME properties and drug-likeness profiles of the non-redundant STF-EAF and STL-EAF metabolites were evaluated using SwissADME. Key descriptors are summarized in Table 4, while the complete SwissADME output is provided in Supplementary Table S22. The analysis revealed variable predicted pharmacokinetic behavior across the metabolites of both fractions.

In STF-EAF, butin (7), 8,3′,4′-trihydroxyflavone (8), and butein (10) showed favorable predicted drug-likeness profiles, including low molecular weight, moderate consensus LogP, acceptable TPSA, no Lipinski violations, high gastrointestinal absorption, and bioavailability scores of 0.55. In contrast, 3,4-dicaffeoylquinic acid (3) showed higher molecular weight, elevated TPSA, increased hydrogen-bonding capacity, three Lipinski violations, low gastrointestinal absorption, and a bioavailability score of 0.11, consistent with its highly polar polyphenolic structure.

For STL-EAF, 9 of the 12 metabolites showed high predicted gastrointestinal absorption and no Lipinski violations, including caffeic acid (1), valine (4), ferulic acid (9), 4′-hydroxy-5,6,7-trimethoxyflavone (11), 3,5,8-trihydroxy-6,7-dimethoxyflavone (12), butin (7), coumarin (14), 7,4′-dihydroxyflavanone (15), and 7,4′-dimethoxyflavone (16). Conversely, naringenin-7-*O*-glucoside (2), maltonic acid (5), and 3,3′,4′,5,5′,7-hexahydroxyflavone (13) showed low predicted gastrointestinal absorption and one Lipinski violation each, likely reflecting their higher polarity and hydrogen-bonding capacity.

All STF-EAF metabolites were predicted to be non-blood–brain barrier (BBB) permeant, whereas ferulic acid, coumarin, 4′,7-dihydroxyflavanone, and selected methoxylated flavones from STL-EAF were predicted to cross the BBB. P-glycoprotein substrate prediction also varied, with 3,4-dicaffeoylquinic acid and butin from STF-EAF, as well as butin and 4′,7-dihydroxyflavanone from STL-EAF, predicted as substrates.

Overall, smaller phenolic acids and flavonoid-type metabolites showed more favorable predicted oral drug-likeness than highly polar glycosylated, polyhydroxylated, or caffeoylquinic acid-derived metabolites, indicating chemically diverse constituents with variable predicted ADME profiles.

### 2.6. Molecular Docking-Based Interaction Analysis

**Figure 10:**
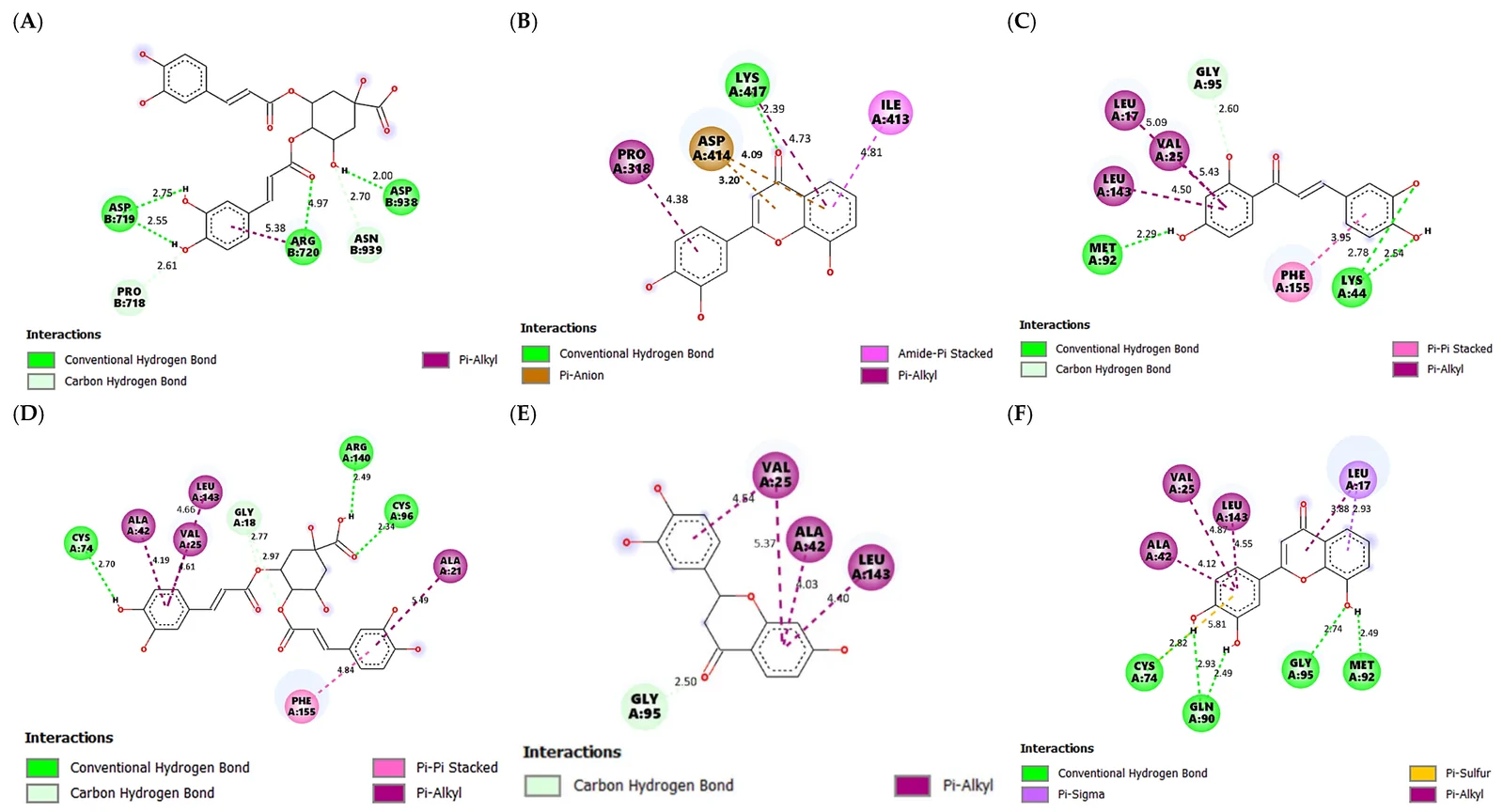

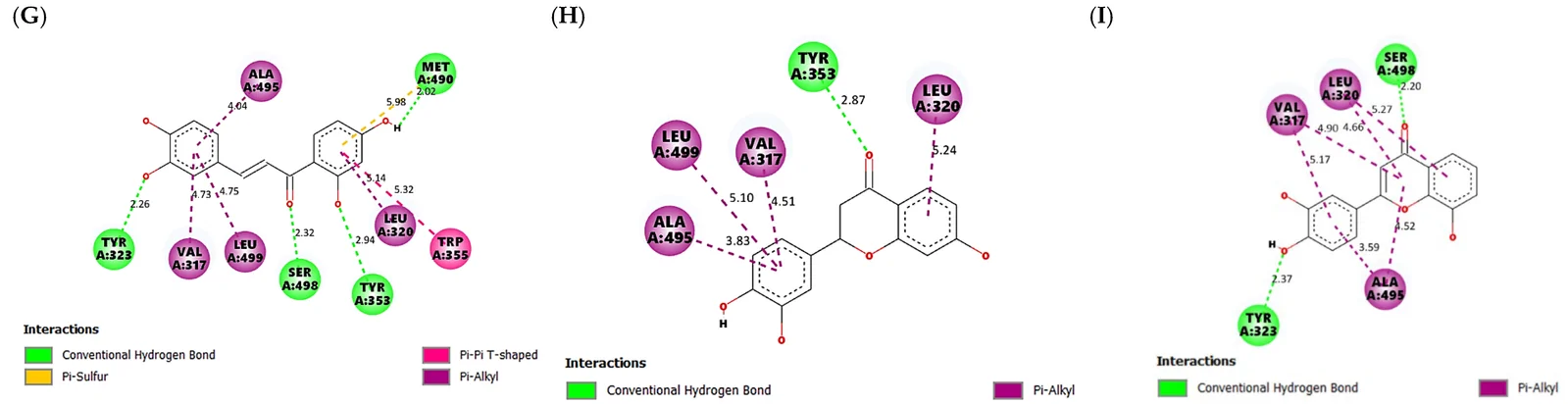
2D interaction maps of selected STF-EAF metabolite–target complexes with docking scores ≤ −7.0 kcal/mol. (**A**) 3,4-Dicaffeoylquinic acid-STAT3; (**B**) 8,3′,4′-trihydroxyflavone-STAT3; (**C**) butein-EGFR; (**D**) 3,4-dicaffeoylquinic acid-EGFR; (**E**) butin-EGFR; (**F**) 8,3′,4′-trihydroxyflavone-EGFR; (**G**) butein-PTGS2; (**H**) butin-PTGS2; (**I**) 8,3′,4′-trihydroxyflavone-PTGS2. STF-EAF, Sphagneticola trilobata flower ethyl acetate fraction.

**Figure 11:**
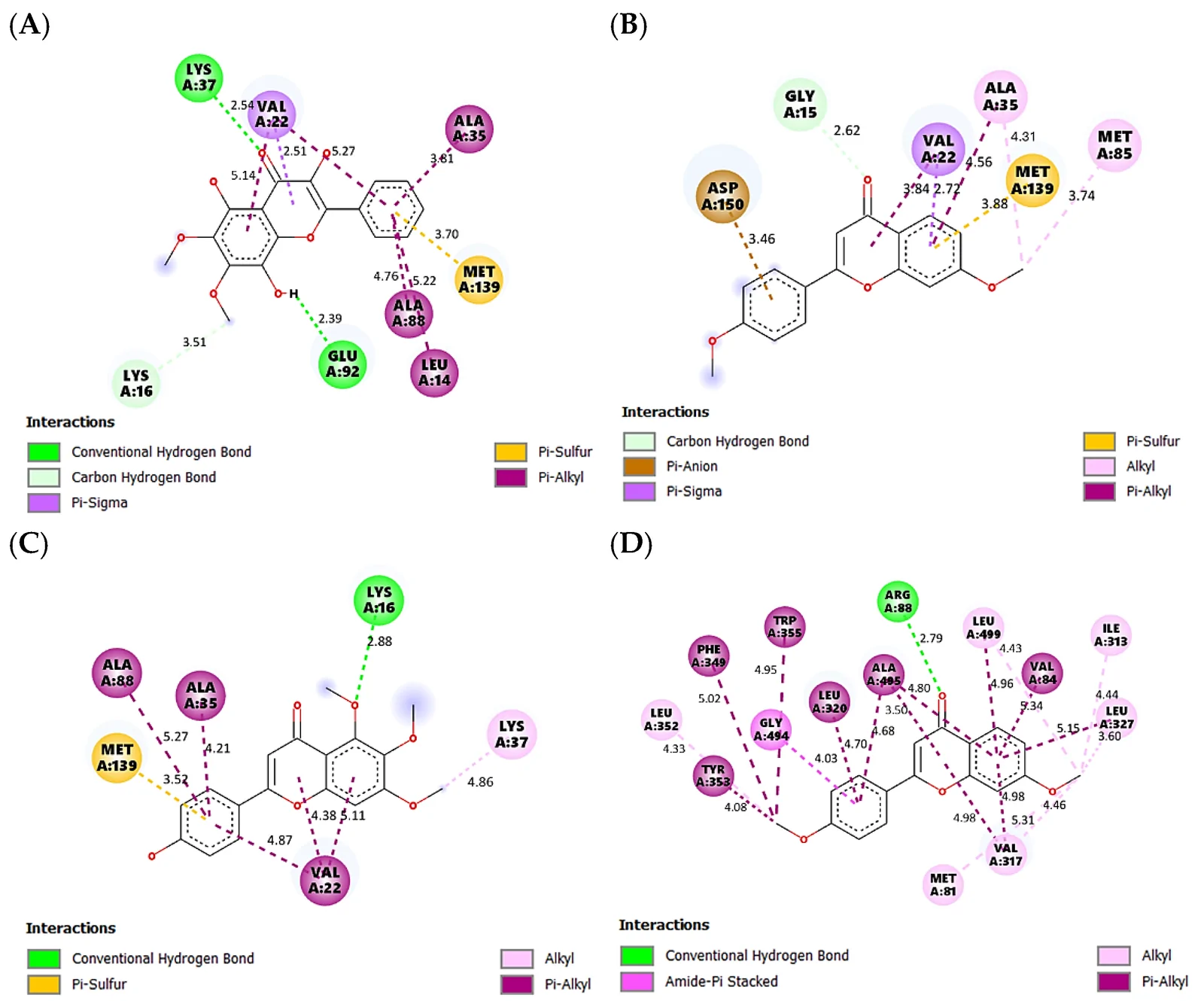
2D maps of selected STL-EAF metabolite–target complexes with docking scores ≤ −7.0 kcal/mol. (**A**) 3,5,8-Trihydroxy-6,7-dimethoxyflavone-AKT1; (**B**) 7,4′-dimethoxyflavone-AKT1; (**C**) 4′-hydroxy-5,6,7-trimethoxyflavone-AKT1; (**D**) 7,4′-dimethoxyflavone-PTGS2. STL-EAF, *S. trilobata* leaf ethyl acetate fraction.

Molecular docking was performed to provide structural support for the network-prioritized targets of STF-EAF and STL-EAF. Docking scores for all tested metabolite–target pairs are summarized in Table 5. Complexes with docking scores ≤ −7.0 kcal/mol and appropriate positioning within the predefined binding region were selected for detailed visualization. Their 2D interaction maps are shown in Figure 10 and Figure 11, while the corresponding 3D binding poses are provided in Supplementary Figures S8 and S9. Detailed interaction parameters, including interaction type, key amino acid residues, and interaction distances, are provided in Supplementary Table S23.

Redocking of the co-crystallized ligands of EGFR, PTGS2, and AKT1 yielded RMSD values of 1.726, 1.852, and 0.397 Å, respectively, with corresponding docking scores of −9.7, −9.1, and −8.8 kcal/mol. All RMSD values were below 2.0 Å, supporting successful reproduction of the crystallographic binding poses.

#### 2.6.1. STF-EAF Docking Profile

For STAT3 (Figure 10A,B), 3,4-dicaffeoylquinic acid and 8,3′,4′-trihydroxyflavone showed the most relevant accommodation within the dimeric STAT3 DNA-binding interface, with docking scores of −7.3 and −7.0 kcal/mol, respectively (Table 5). Butein and butin showed lower scores of −6.9 and −6.8 kcal/mol, respectively. The best-ranked pose of 3,4-dicaffeoylquinic acid interacted mainly with chain B residues ASP719, ARG720, and ASP938, with additional contacts involving PRO718 and ASN939. In contrast, 8,3′,4′-trihydroxyflavone occupied the corresponding chain A interface region, forming a conventional hydrogen bond with LYS417 and *π*-associated interactions with ASP414, ILE413, and PRO318. These patterns support the accommodation of selected STF-EAF metabolites within the STAT3 DNA-binding interface (Supplementary Table S23).

For EGFR (Figure 10C–F), all selected STF-EAF metabolites were accommodated within the ATP-binding pocket, with docking scores ranging from −9.0 to −7.7 kcal/mol (Table 5). 3,4-Dicaffeoylquinic acid showed the highest score (−9.0 kcal/mol), stabilized by hydrogen bonds with CYS74(A), ARG140(A), and CYS96(A), together with *π*-associated contacts involving PHE155(A), ALA21(A), ALA42(A), VAL25(A), and LEU43(A). 8,3′,4′-Trihydroxyflavone also showed favorable accommodation (−8.0 kcal/mol), forming hydrogen bonds with CYS74(A), GLN90(A), GLY95(A), and MET92(A), and *π*-associated contacts with CYS74(A), LEU17(A), VAL25(A), ALA42(A), and LEU143(A). Butein and butin showed comparable scores of −7.8 and −7.7 kcal/mol, respectively, with poses mainly supported by hydrogen-bonding and hydrophobic interactions within the same ATP-binding pocket (Supplementary Table S23).

For PTGS2 (Figure 10G–I), flavonoid-type metabolites showed the most favorable accommodation within the cyclooxygenase channel. 8,3′,4′-Trihydroxyflavone showed the highest score (−8.8 kcal/mol), followed by butin (−8.5 kcal/mol) and butein (−7.8 kcal/mol), whereas 3,4-dicaffeoylquinic acid showed a lower score (−6.3 kcal/mol) (Table 5). The pose of 8,3′,4′-trihydroxyflavone was supported by hydrogen bonds with SER498(A) and TYR323(A), and *π*-alkyl contacts with VAL317(A), LEU320(A), and ALA495(A). Butin formed a hydrogen bond with TYR353(A) and hydrophobic contacts with LEU499(A), VAL317(A), ALA495(A), and LEU320(A). Butein occupied the same channel through hydrogen bonds with TYR323(A), SER498(A), TYR353(A), and MET490(A), in addition to *π*-associated contacts involving MET490(A), TRP355(A), ALA495(A), VAL317(A), LEU499(A), and LEU320(A). Overall, these results provide structural support for the interaction of STF-EAF flavonoid-type metabolites, particularly 8,3′,4′-trihydroxyflavone and butin, with PTGS2 (Supplementary Table S23).

#### 2.6.2. STL-EAF Docking Profile

For AKT1 (Figure 11A–C), docking was performed within the ATP-binding pocket defined by the co-crystallized ATP-competitive inhibitor. Among STL-EAF metabolites, 3,5,8-trihydroxy-6,7-dimethoxyflavone showed the highest AKT1 score (−8.0 kcal/mol), followed by 4′-hydroxy-5,6,7-trimethoxyflavone and 7,4′-dimethoxyflavone, each with −7.6 kcal/mol. Caffeic acid and ferulic acid showed lower scores of −6.8 and −6.4 kcal/mol, respectively (Table 5).

The best-ranked pose of 3,5,8-trihydroxy-6,7-dimethoxyflavone was stabilized by conventional hydrogen bonds with LYS37(A) and GLU92(A), a carbon hydrogen bond with LYS16(A), and π-associated/hydrophobic contacts with VAL22(A), LEU14(A), ALA35(A), ALA88(A), and MET139(A). 4′-Hydroxy-5,6,7-trimethoxyflavone formed a hydrogen bond with LYS16(A), a π-sulfur interaction with MET139(A), and hydrophobic contacts with VAL22(A), ALA35(A), ALA88(A), and LYS37(A). 7,4′-Dimethoxyflavone formed a carbon hydrogen bond with GLY15(A), a π-anion interaction with ASP150(A), and additional π-associated/hydrophobic contacts with VAL22(A), MET139(A), ALA35(A), and MET85(A). These results indicate more favorable AKT1 pocket accommodation of STL-EAF flavone-type metabolites than the smaller phenolic acids (Supplementary Table S23).

Docking of STL-EAF metabolites at the STAT3 DNA-binding interface produced modest scores ranging from −5.5 to −6.8 kcal/mol. 7,4′-Dimethoxyflavone showed the highest score (−6.8 kcal/mol), followed by 3,5,8-trihydroxy-6,7-dimethoxyflavone (−6.5 kcal/mol), 4′-hydroxy-5,6,7-trimethoxyflavone (−6.4 kcal/mol), caffeic acid (−5.7 kcal/mol), and ferulic acid (−5.5 kcal/mol) (Table 5). These results provide limited docking support for direct accommodation of STL-EAF metabolites within the selected STAT3 interface, suggesting that STAT3 is better interpreted as a network-predicted signaling hub under the applied docking conditions (Supplementary Table S23).

For PTGS2 (Figure 11D), docking was performed within the mefenamic acid-defined cyclooxygenase channel. Among STL-EAF metabolites, 7,4′-dimethoxyflavone showed the highest PTGS2 score (−7.9 kcal/mol), whereas ferulic acid, caffeic acid, 3,5,8-trihydroxy-6,7-dimethoxyflavone, and 4′-hydroxy-5,6,7-trimethoxyflavone showed lower scores of −6.9, −6.6, −6.6, and −6.3 kcal/mol, respectively (Table 5). The 7,4′-dimethoxyflavone pose was stabilized by a conventional hydrogen bond with ARG88, an amide-π stacked interaction with GLY494, and multiple alkyl/π-alkyl contacts involving LEU320, ALA495, LEU499, VAL84, VAL317, LEU327, ILE313, and MET81 (Supplementary Table S23).

The docking analysis highlighted a fraction-specific pattern of target accommodation. STF-EAF metabolites showed stronger structural compatibility with EGFR and PTGS2, with additional support at the STAT3 DNA-binding interface, whereas STL-EAF metabolites showed clearer docking support for AKT1 and PTGS2, mainly through flavone-type metabolites. Collectively, these findings reinforce the network pharmacology results by providing structure-based support for selected metabolite–target interactions and prioritizing them for future experimental validation.

## 3. Discussion

The present study provides an integrated phytochemical, biological, and computational evaluation of the antioxidant potential of *Sphagneticola trilobata* flowers and leaves. By combining UPLC–ESI–MS/MS profiling, antioxidant assays, network pharmacology, ADME prediction, and molecular docking, the study offers a multi-level interpretation of the active fractions [15,69]. This strategy is particularly suitable for natural product fractions, where biological activity commonly reflects the collective contribution of multiple metabolites rather than the effect of a single compound [15]. Accordingly, the antioxidant potential of *S. trilobata* can be interpreted through both direct chemical antioxidant capacity and predicted modulation of redox-sensitive molecular targets and pathways.

UPLC–ESI–MS/MS profiling revealed marked solvent- and organ-dependent variation in the metabolite composition of *S. trilobata*. The petroleum ether fractions showed the broadest chemical diversity and were mainly enriched in lipophilic constituents, including ent-kaurane diterpenes, fatty acids and their derivatives, triterpenes, sterols, coumarins, and related metabolites. The methylene chloride fractions contained a mixed profile of flavonoids, phenolic acids, diterpenoid-related metabolites, fatty acids, coumarins, and glycosidic constituents. In contrast, the ethyl acetate fractions were characterized by a more polar phenolic and flavonoid-rich profile. The flower ethyl acetate fraction contained butein, butin, 3,4-dicaffeoylquinic acid, a dicaffeoylquinic acid isomer, and 8,3′,4′-trihydroxyflavone, whereas the leaf ethyl acetate fraction contained caffeic acid, ferulic acid, naringenin-7-*O*-glucoside, butin, 7,4′-dihydroxyflavanone, and methoxylated flavones. This phenolic/flavonoid-rich composition supports the relevance of the ethyl acetate fractions for subsequent antioxidant and in silico mechanistic investigations.

The LC–MS/MS data supported tentative candidate-level annotation of the detected metabolites. Differences in retention time and product-ion profiles further supported the presence of distinct diterpenoid-related chromatographic features, although definitive differentiation of closely related positional and stereochemical isomers requires authentic reference standards or complementary high-resolution MS and spectroscopic analyses.

The isolation of butein from the flower ethyl acetate fraction was consistent with its predominance in this fraction based on relative abundance. Butein is a chalcone-type flavonoid characterized by a conjugated aromatic system and multiple hydroxyl groups, structural features commonly associated with electron donation, hydrogen atom transfer, and radical stabilization [70,71]. However, the flower ethyl acetate fraction showed stronger antioxidant activity than isolated butein in both ABTS and FRAP assays. This finding indicates that butein is an important contributor to the activity of the fraction but does not fully account for its antioxidant potency. The superior activity of the fraction relative to both isolated butein and ascorbic acid suggests that its antioxidant response reflects the combined contribution of multiple constituents rather than the action of a single predominant metabolite. Co-occurring phenolic and flavonoid-type metabolites, including butin, dicaffeoylquinic acid derivatives, and trihydroxyflavone derivatives, are structurally diverse, with differences in hydroxylation pattern, degree of conjugation, polarity, and redox behavior. These differences may provide complementary radical-scavenging and electron-donating effects across the ABTS and FRAP assays [72]. The resulting interaction may be additive or potentially synergistic [73,74]; however, definitive synergy cannot be established from the present experiments and would require evaluation of defined metabolite combinations using appropriate interaction analyses.

The antioxidant assays confirmed the biological relevance of the phytochemical findings. The ethyl acetate flower fraction showed the strongest activity in both ABTS and FRAP assays, followed by the ethyl acetate leaf fraction, whereas the petroleum ether leaf fraction was the least active among the tested samples. Since ABTS reflects radical scavenging capacity and FRAP reflects reducing power, the parallel activity pattern suggests that the ethyl acetate fractions possess both radical-quenching and electron-donating properties. According to the IC_50_-based antioxidant classification reported by Fucina et al. (2016) [61] and Mustarichie et al. (2017) [75], samples with IC_50_ values below 50 µg/mL are considered very potent antioxidants. Based on this classification, the ethyl acetate flower fraction, the ethyl acetate leaf fraction, and isolated butein showed very potent ABTS radical scavenging activity. The ethyl acetate flower and leaf fractions also showed very potent reducing activity in the FRAP assay, further confirming their antioxidant efficiency.

The superior activity of the ethyl acetate flower fraction is chemically consistent with its phenolic and flavonoid-rich profile. Quinic acid derivatives have been previously associated with antioxidant activity [76], while polyphenolic compounds, flavonoids, and quinone-related metabolites have been widely reported as effective contributors to free radical scavenging and reducing capacity [75,77]. This interpretation is further supported by a recent comparative metabolomics study on *S. trilobata* flowerheads and leaves, which reported that flowerheads were enriched in flavonoids and phenolic acids and exhibited stronger antioxidant activity than leaves, whereas leaves were more characterized by terpenoid abundance [13]. Additional pharmacological studies have also reported antioxidant or related bioactivities for different extracts and organs of *S. trilobata*, including flower extracts evaluated using DPPH radical scavenging and tyrosinase inhibition assays [78,79,80]. Collectively, these reports support the antioxidant relevance of *S. trilobata* and are consistent with the enrichment of its flower-derived preparations in polyphenolic constituents, which are widely recognized for their free-radical scavenging capacity.

Network pharmacology was subsequently applied to extend the interpretation beyond direct chemical antioxidant behavior and to explore the possible biological targets and pathways associated with the active ethyl acetate fractions. The curated metabolites of the flower ethyl acetate fraction (STF-EAF) and leaf ethyl acetate fraction (STL-EAF) were predicted to interact with 61 and 82 oxidative stress-related targets, respectively. The broader target coverage of STL-EAF may be related to its larger curated metabolite set, whereas the stronger in vitro antioxidant activity of STF-EAF indicates that antioxidant potency is not determined solely by the number of predicted targets. This distinction highlights the complementary value of the experimental and computational approaches: ABTS and FRAP demonstrate direct antioxidant capacity, whereas network pharmacology suggests possible engagement of redox-sensitive molecular systems.

The compound-target networks supported the multi-component nature of both active fractions. In STF-EAF, butein showed the highest degree centrality, while 3,4-dicaffeoylquinic acid, butin, and 8,3′,4′-trihydroxyflavone displayed comparable target coverage. This finding agrees with the experimental observation that the whole flower ethyl acetate fraction was more active than isolated butein, supporting the contribution of several constituents rather than a single major metabolite. In STL-EAF, ferulic acid, caffeic acid, and methoxylated flavones showed high predicted connectivity, indicating that both phenolic acids and flavone-type metabolites may contribute to the predicted antioxidant-related target network. These results support a multi-compound, multi-target model for the antioxidant activity of *S. trilobata* ethyl acetate fractions.

PPI network analysis further identified key redox-sensitive hub proteins that may underlie the predicted biological relevance of both fractions. Shared or highly ranked targets, including STAT3, JUN, CTNNB1, EGFR, PTGS2, BCL2, MMP9, ESR1, and TLR4, are functionally linked to inflammatory signaling, apoptosis regulation, transcriptional control, cell survival, and oxidative stress responses. This is consistent with previous reports describing the involvement of STAT3 redox regulation, NF-κB/AP-1-related inflammatory transcriptional signaling, EGFR redox-sensitive kinase signaling, and PTGS2/COX2-mediated inflammatory responses in oxidative stress-associated cellular regulation [81,82,83,84,85]. STL-EAF additionally prioritized AKT1, NFKB1, APP, CCND1, and PPARG, suggesting broader predicted involvement in survival signaling, inflammatory transcriptional regulation, cell-cycle control, and metabolic stress responses [83,86,87,88,89]. These targets provide biologically meaningful candidates for future mechanistic validation.

Functional enrichment analysis reinforced the network-derived interpretation. Both fractions were enriched in biological processes and pathways associated with inflammation, apoptosis regulation, transcriptional control, hypoxia response, lipid-associated stress, and kinase-mediated signaling. STF-EAF showed stronger association with MAPK cascade, cytokine-mediated signaling, IL-17 signaling, and C-type lectin receptor signaling, whereas STL-EAF was additionally characterized by nitric oxide biosynthesis, xenobiotic and UV-A responses, fluid shear stress, focal adhesion, and relaxin-related signaling. These enrichment patterns suggest that both fractions share a core redox-regulatory framework while retaining fraction-specific mechanistic features. The recurrence of inflammation-, apoptosis-, hypoxia-, and lipid-associated pathways is consistent with the central role of oxidative stress in coordinating cellular defense, inflammatory responses, cell fate regulation, metabolic adaptation, and stress-response signaling [90,91,92].

ADME profiling added a pharmacokinetic prioritization layer to the mechanistic interpretation, as SwissADME provides predictive descriptors related to physicochemical properties, gastrointestinal absorption, BBB permeability, P-glycoprotein substrate status, drug-likeness, and bioavailability [93]. Smaller phenolic acids and flavonoid-type metabolites, including butin, butein, caffeic acid, ferulic acid, coumarin, 4′,7-dihydroxyflavanone, and selected methoxylated flavones, showed more favorable predicted oral drug-likeness, including high gastrointestinal absorption and limited or no Lipinski violations. These properties increase the pharmacokinetic plausibility of their contribution to systemic antioxidant-related effects, particularly because most of these metabolites were also predicted not to be P-glycoprotein substrates. In contrast, highly polar metabolites, such as 3,4-dicaffeoylquinic acid, naringenin-7-*O*-glucoside, maltonic acid, and 3,3′,4′,5,5′,7-hexahydroxyflavone, showed less favorable predicted absorption profiles, mainly due to increased polarity and hydrogen-bonding capacity. These properties may reduce systemic bioavailability and consequently limit the extent to which these metabolites can reach distant molecular targets. These trends are consistent with recent analyses showing that molecular size, lipophilicity, polarity, and hydrogen-bonding properties remain useful descriptors for oral drug-likeness assessment, although they should be applied as prioritization tools rather than rigid exclusion rules [94,95]. Nevertheless, less absorbable polar metabolites may still contribute to the overall antioxidant activity of the fractions through direct radical scavenging, local gastrointestinal effects, metabolic transformation, or interaction with other constituents. Overall, SwissADME predictions provide a useful basis for metabolite prioritization while preserving the potential relevance of polar constituents within an extract-based context. However, these predictions do not establish actual in vivo exposure or efficacy and require confirmation through experimental permeability, metabolic stability, and pharmacokinetic studies.

Molecular docking provided a structure-based layer that refined the network pharmacology findings by identifying metabolite–target pairs with favorable accommodation within functionally relevant binding regions. For STF-EAF, the docking profile showed strong structural compatibility with EGFR and PTGS2, with additional support for selected metabolites at the STAT3 DNA-binding interface. 3,4-Dicaffeoylquinic acid showed the most favorable docking score within the EGFR ATP-binding pocket, whereas 8,3′,4′-trihydroxyflavone, butin, and butein showed favorable accommodation within the PTGS2 cyclooxygenase channel. These results suggest that STF-EAF metabolites, particularly flavonoid- and chalcone-type constituents, may contribute to the predicted redox-related network through structurally plausible interactions with kinase and inflammatory targets. The interface-directed docking of selected metabolites with STAT3 further supports the relevance of STAT3 as a redox-sensitive transcriptional hub within the predicted mechanism.

For STL-EAF, the strongest docking support was observed for AKT1 and PTGS2, mainly through flavone-type metabolites. Methoxylated flavones, including 3,5,8-trihydroxy-6,7-dimethoxyflavone, 7,4′-dimethoxyflavone, and 4′-hydroxy-5,6,7-trimethoxyflavone, showed favorable accommodation within the AKT1 ATP-binding pocket, whereas 7,4′-dimethoxyflavone showed the most relevant PTGS2 interaction. In contrast, STL-EAF metabolites showed weaker docking support at the STAT3 DNA-binding interface, suggesting that STAT3 may function primarily as a network-supported regulatory hub for this fraction, while AKT1 and PTGS2 receive stronger structure-based support. Thus, the docking analysis revealed a fraction-specific pattern of target compatibility, with STF-EAF preferentially supporting EGFR/PTGS2 and selected STAT3-related interactions, and STL-EAF preferentially supporting AKT1/PTGS2 interactions.

Taken together, the findings demonstrate the direct radical-scavenging and reducing activities of the *S. trilobata* ethyl acetate fractions and suggest the possible involvement of redox-sensitive pathways based on the computational analyses. The phenolic- and flavonoid-rich profile, particularly in the flower ethyl acetate fraction, was consistent with the strong ABTS and FRAP activities, whereas network pharmacology, ADME prediction, and molecular docking provided a framework for prioritizing candidate metabolites, targets, and pathways for further investigation.

Beyond these mechanistic priorities, future studies should examine the residual hydroethanolic fraction and extend the investigation to other plant organs and a wider range of extraction solvents with different polarities. Broader biological evaluation of the resulting extracts may provide a more comprehensive appraisal of the phytochemical diversity and pharmacological potential of *S. trilobata*.

In parallel, the proposed target–pathway associations and ligand–protein interactions have yet to be confirmed experimentally, and their validation would further strengthen the mechanistic interpretation of the integrated findings. Cellular oxidative stress models, intracellular ROS measurements, antioxidant enzyme assays, mitochondrial stress markers, target-specific biochemical assays, and gene/protein expression analyses of prioritized hubs such as STAT3, EGFR, PTGS2, AKT1, NFKB1, and TLR4 would help substantiate these associations and clarify the contributions of additive, cooperative, and target-directed effects.

## 4. Materials and Methods

### 4.1. Plant Material and Extracts Preparation

In July 2022, 250 g of *S. trilobata* flowers and 3 kg of leaves were obtained from Cairo Festival City Mall, Cairo, Egypt. Botanical authentication was performed by Prof. Abd El-Halim Abd El-Mogali Mohamed of the Flora and Phytotaxonomy Research Department, Horticultural Research Institute, Agricultural Research Centre, Agricultural Museum, Dokki, Giza, Egypt. A reference sample was archived under voucher number ST-Co 15 at the Herbarium, Faculty of Pharmacy, Zagazig University, Egypt.

After washing and air-drying, the plant materials were ground and macerated three successive times with fresh 70% ethanol at room temperature for 10 days per cycle. The initial plant-to-solvent ratios were 1:20 and 1:5 (*w*/*v*) for the flowers and leaves, respectively; the solvent volumes used were 5, 3, and 2.5 L for the flower cycles and 15, 10, and 7 L for the leaf cycles. Concentration under reduced pressure produced 50 g of flower extract and 350 g of leaf extract, corresponding to yields of 20.00% and 11.67%, respectively. Of these, 30 and 300 g, respectively, were sequentially partitioned with petroleum ether, methylene chloride, and ethyl acetate using 6 × 250, 12 × 250, and 16 × 250 mL for the flower extract and 18 × 500, 20 × 500, and 20 × 500 mL for the leaf extract, respectively. The flower fractions yielded 3, 4, and 3 g (10.00%, 13.33%, and 10.00%), whereas the leaf fractions yielded 20, 10, and 4.5 g (6.67%, 3.33%, and 1.50%), respectively. The dried extracts and fractions were stored in amber glass bottles in a freezer until use.

### 4.2. Chemicals and Reagents

All chemicals and reagents were obtained from Sigma-Aldrich, Hamburg, Germany. These included HPLC-grade methanol (≥99.9%), acetonitrile, ESI-MS-grade water, reagent-grade formic acid (≥95%), nordihydroguaiaretic acid (NDGA), and ascorbic acid (99%). Silica gel GF254, Sephadex LH-20, TLC plates, and the solvents employed in chromatographic separation and TLC analysis were of analytical grade.

### 4.3. UPLC–ESI–MS/MS Chemical Profiling

UPLC–ESI–MS/MS analysis was conducted in positive and negative ionization modes using a XEVO TQD triple–quadrupole mass spectrometer (Waters Corporation, Milford, MA, USA). Quantitative phytochemical analysis was carried out in multiple-reaction monitoring (MRM) mode. The flower and leaf fractions of *S. trilobata* were examined to establish their chromatographic profiles. Each fraction was dissolved in HPLC-grade methanol, passed through a 0.2 μm membrane filter, and adjusted to a final concentration of 0.2–0.5 mg/mL according to the fraction analyzed.

Chromatographic separation was achieved on an ACQUITY UPLC BEH C18 column (2.1 × 50 mm, 1.7 μm; Waters Corporation, Milford, MA, USA) at 0.2 mL/min using a previously described gradient method [96]. The mobile phases were water containing 0.1% formic acid (A) and methanol containing 0.1% formic acid (B). The gradient started with 10% B for 0–2 min, increased linearly to 30% B from 2–5 min, to 70% B from 5–15 min, and to 90% B from 15–22 min. Isocratic elution at 90% B was then maintained from 22–25 min, followed by column washing and re-equilibration.

To maximize spectral coverage, electrospray ionization was applied in both polarity modes. In negative mode, the operating conditions were: source temperature, 150 °C; cone voltage, 30 eV; capillary voltage, 3 kV; desolvation temperature, 440 °C; cone gas flow, 50 L/h; and desolvation gas flow, 900 L/h. Spectra were recorded over an *m/z* range of 100–1000. Metabolite annotations were based on precursor and product ions, diagnostic neutral losses, chromatographic behavior, and comparison of the observed MS/MS fragmentation patterns with published data. Data processing was performed using MassLynx 4.1. In the absence of authentic reference standards, most assignments were regarded as tentative candidate structures, broadly corresponding to Level 3 of the Schymanski confidence framework [97]. Closely related positional and stereochemical isomers could not be unambiguously differentiated under the applied analytical conditions; however, differences in retention time and fragmentation profiles supported the presence of distinct chromatographic features.

### 4.4. Chromatographic Isolation of the Predominant Compound

A 3 g portion of the *S. trilobata* flower ethyl acetate fraction was chromatographed over silica gel (60–230 mesh) in a 100 × 1 cm glass column. Elution began with *n*-hexane, after which the solvent polarity was progressively increased with dichloromethane and subsequently methanol. The collected fractions were examined by TLC on silica gel GF_254_ plates using benzene:ethyl acetate:acetic acid (4:5:0.5, *v*/*v*/*v*), and fractions showing comparable chromatographic patterns were pooled. Fractions 30–39, obtained with CH_2_Cl_2_: MeOH (99:1, *v*/*v*), were further separated on a Sephadex LH-20 column (30 × 1.5 cm) using methanol, affording 150 mg of the isolated compound as orange-yellow needles. TLC analysis of the purified compound with benzene: ethyl acetate: acetic acid (5:3.5:0.5, *v*/*v*/*v*) gave an R_f_ value of 0.58.

### 4.5. Spectroscopic Characterization of the Isolated Compound

Structural characterization of the isolated compound was carried out by UV spectroscopy, ESI-MS/MS, ^1^H-NMR, ^13^C-NMR, HSQC, and HMBC analyses. UV spectra were obtained in methanol on a Shimadzu UV-1700 spectrophotometer (Shimadzu Corporation, Kyoto, Japan), followed by shift-reagent studies with NaOMe, AlCl_3_, AlCl_3_/HCl, NaOAc, and NaOAc/H_3_BO_3_. Negative-mode ESI-MS/MS measurements were performed using the UPLC–ESI–MS/MS system detailed in Section 4.3. NMR spectra were acquired in CD_3_OD on a Bruker spectrometer (Bruker, Switzerland) at 400 MHz for ^1^H-NMR and 100 MHz for ^13^C-NMR. Chemical shifts were expressed as δ values in ppm, while coupling constants were reported in Hz. Structure elucidation was based on the combined interpretation of UV, MS, 1D-NMR, and 2D-NMR data, together with comparison to the published literature.

### 4.6. Evaluation of Antioxidant Activity

Antioxidant potential was assessed at the Regional Center for Mycology and Biotechnology (RCMB), Al-Azhar University, by ABTS radical-scavenging and FRAP methods. Each assay was conducted in triplicate, and the data were presented as mean ± SD.

#### 4.6.1. ABTS [2,2′-Azinobis(3-ethylbenzothiazoline-6-sulphonic acid)] Free Radical Scavenging Activity Assay

The ABTS method is based on the reduction in color intensity resulting from the reaction between antioxidants and chemically generated ABTS radicals [98]. The assay followed previously published protocols [99,100]. The ABTS radical cation (ABTS•+) was generated by mixing 1.8 mM ABTS stock solution (Sigma, PN: A3219) with 0.63 mM potassium persulfate, followed by incubation in the dark at room temperature for 12–16 h. Before use, the solution was diluted with ethanol until an absorbance of 0.700 ± 0.030 at 734 nm was reached. Samples were diluted tenfold with 80% methanol. In each well of a microtiter plate, 10 μL of the diluted sample was combined with 190 μL of ABTS•+ solution. Absorbance at 734 nm was recorded at 1 min intervals for 13 min. Solvent blanks were included, while ascorbic acid and methanol served as the reference antioxidant and negative control, respectively. Radical-scavenging activity was calculated using the following equation:

% Free radical scavenging activity = [(A_n_ − A_s_)/A_n_] × 100

where A_n_ represents the final absorbance of the negative control and A_s_ denotes the final absorbance of the sample.

#### 4.6.2. Ferric Reducing Antioxidant Power (FRAP) Assay

The reducing ability of the samples was determined using the FRAP assay, which reflects antioxidant potential through the conversion of ferric ions to ferrous ions, following the methods of Oyaizu (1986) [101] and Ferreira et al. (2007) [102]. Each sample, dissolved in 1 mL methanol, was combined with 2.5 mL of 0.2 M sodium phosphate buffer (pH 6.6) and 2.5 mL of 1% (*w*/*v*) potassium ferricyanide [K_3_Fe(CN)_6_]. The reaction mixture was incubated at 50 °C for 20 min, then treated with 2.5 mL of 10% (*w*/*v*) trichloroacetic acid and centrifuged at 1000× *g* for 10 min. A 2.5 mL aliquot of the supernatant was mixed with 2.5 mL deionized water and 0.5 mL of freshly prepared 0.1% (*w*/*v*) ferric chloride. Absorbance was recorded at 700 nm against a blank using a Milton Roy Spectronic 1201 spectrophotometer. Ascorbic acid served as the reference antioxidant, and higher absorbance values indicated stronger reducing capacity. Data were expressed as mean ± SD.

#### 4.6.3. Statistical Analysis

Results were presented as mean ± SD. Each concentration in the ABTS and FRAP assays was measured three times within the same experiment. ABTS and FRAP data were analyzed by two-way ANOVA followed by Bonferroni’s multiple-comparisons test. Statistical significance was accepted at *p* < 0.05. IC_50_ values were determined from the corresponding concentration–response curves by nonlinear regression using GraphPad Prism version 5.0 (GraphPad Software, San Diego, CA, USA) and reported in µg/mL.

### 4.7. Network Pharmacology Analysis

#### 4.7.1. Metabolite Curation and Target Prediction

Network pharmacology analysis was performed for STF-EAF and STL-EAF, respectively. The LC–MS/MS-identified metabolites of each fraction were curated independently before target prediction. Duplicated entries and repeated isomeric annotations were removed, and only metabolites with defined chemical structures were retained. Canonical SMILES were retrieved from PubChem (https://pubchem.ncbi.nlm.nih.gov; accessed 5 June 2026) [103]. For metabolites without available PubChem records, the proposed structures were drawn using ChemDraw software v22.0.0.22 (PerkinElmer Informatics, Inc., High Wycombe, UK), and the corresponding SMILES were generated.

The SMILES of each curated metabolite were submitted individually to SwissTargetPrediction (http://www.swisstargetprediction.ch/; accessed 5 June 2026) [104], using *Homo sapiens* as the target organism. All predicted targets returned by SwissTargetPrediction had non-zero probability values and were retained for subsequent analysis. These targets were standardized to official gene symbols using UniProt (https://www.uniprot.org/; accessed 6 June 2026) [105], and duplicated targets were removed to generate non-redundant target sets for STF-EAF and STL-EAF.

#### 4.7.2. Oxidative Stress-Related Target Collection and Intersection Analysis

Oxidative stress-related targets were collected from GeneCards (https://www.genecards.org/; accessed 6 June 2026) [106] and the Online Mendelian Inheritance in Man database (OMIM; https://www.omim.org/; accessed 6 June 2026) [107], using “oxidative stress” as the search term. GeneCards-derived targets were filtered using a relevance score cutoff of ≥10 to retain genes with stronger relevance to oxidative stress. The retrieved targets were standardized to official human gene symbols, merged, and deduplicated to generate the final oxidative stress-related target set.

The predicted targets of STF-EAF and STL-EAF were separately intersected with the oxidative stress-related target set using Venny 2.1.0 (https://bioinfogp.cnb.csic.es/tools/venny/; accessed 6 June 2026). The overlapping targets were retained as putative antioxidant-relevant targets and were used for subsequent PPI network construction, compound-target network analysis, and functional enrichment.

#### 4.7.3. PPI Network Construction and Topological Analysis

The antioxidant-relevant overlapping targets of each fraction were submitted independently to STRING v12.0 (https://string-db.org/; accessed 7 June 2026) [108] for PPI network construction. *Homo sapiens* was selected as the organism, and the minimum required interaction score was set to 0.7, corresponding to high-confidence interactions. Disconnected nodes were hidden before downstream topological analysis.

The STRING-derived PPI networks were imported into Cytoscape v3.10.2 (NIGMS, USA) for visualization and network analysis [109]. CytoNCA was used to calculate degree centrality (DC), betweenness centrality (BC), and closeness centrality (CC). Hub targets were identified using a two-step median-based topological filtering strategy. First, nodes with DC values greater than or equal to the median DC of the initial network were retained to generate a primary subnetwork. Second, this subnetwork was further filtered using the median values of DC, BC, and CC. Nodes satisfying these criteria were considered core hub targets for each fraction.

#### 4.7.4. Compound-Target Network Construction and Functional Enrichment Analysis

Compound-target networks were constructed separately for STF-EAF and STL-EAF using Cytoscape v3.10.2 to visualize the associations between the curated metabolites and their corresponding antioxidant-relevant targets. Nodes were classified as compounds or protein targets, and DC was used to rank metabolites according to their predicted target connectivity.

Core hub targets were subjected to functional enrichment analysis using the Database for Annotation, Visualization and Integrated Discovery (DAVID; https://davidbioinformatics.nih.gov/; accessed 8 June 2026) [110], with *Homo sapiens* specified as the reference organism. Gene Ontology enrichment covered the biological process (BP), cellular component (CC), and molecular function (MF) domains, while pathway enrichment was assessed using the Kyoto Encyclopedia of Genes and Genomes (KEGG). GO terms and KEGG pathways with Benjamini-adjusted *p*-values ≤ 0.05 were regarded as significant and retained for biological interpretation.

### 4.8. In Silico ADME and Drug-likeness Profiling

In silico absorption, distribution, metabolism, and excretion (ADME) properties and drug-likeness parameters of the non-redundant metabolites retained from STF-EAF and STL-EAF were evaluated using SwissADME (http://www.swissadme.ch/; accessed 9 June 2026) [93]. The canonical SMILES of each metabolite were submitted individually. The evaluated descriptors included physicochemical properties, Lipinski’s rule of five parameters, topological polar surface area (TPSA), lipophilicity, water solubility, gastrointestinal absorption, blood–brain barrier permeability, P-glycoprotein substrate status, and bioavailability score.

ADME profiling was used as a supportive pharmacokinetic annotation and prioritization layer rather than as a strict exclusion criterion, considering the extract-based nature of the study and the potential contribution of metabolites with diverse physicochemical properties to the overall antioxidant activity.

### 4.9. Molecular Docking Analysis

Molecular docking was performed as a structure-based follow-up to the network pharmacology analysis to evaluate the binding plausibility of selected STF-EAF and STL-EAF metabolites against prioritized antioxidant-related targets. Target prioritization was performed among the fraction-specific core hub targets retained after PPI topological filtering, considering their centrality, involvement in significantly enriched redox-related pathways, the literature-supported relevance to oxidative stress, and the availability of experimentally resolved structures with defined binding regions suitable for docking. Based on these combined criteria, STAT3, EGFR, and PTGS2 were selected for STF-EAF, whereas AKT1, STAT3, and PTGS2 were selected for STL-EAF. The docked metabolites were selected from the top-ranked compounds in the compound-target networks according to degree centrality.

The crystal structures were retrieved from the RCSB Protein Data Bank (https://www.rcsb.org; accessed 12 June 2026) [111]. The selected structures were STAT3 (PDB ID: 6QHD) [112], EGFR (PDB ID: 5U8L) [113], PTGS2 (PDB ID: 5IKR) [114], and AKT1 (PDB ID: 4GV1) [115]. For STAT3, the DNA-bound structure was used to define the DNA-binding interface, and the DNA molecule was removed before docking. For EGFR, PTGS2, and AKT1, the co-crystallized ligands were used to define the corresponding binding pockets and were subsequently removed. Chain A was retained for EGFR, PTGS2, and AKT1, whereas the dimeric STAT3 assembly was retained to preserve the DNA-binding interface.

Protein preparation was performed using UCSF Chimera v1.17.3 [116] by removing water molecules, non-essential heteroatoms, and co-crystallized ligands as appropriate, followed by hydrogen atom addition. Ligand structures were retrieved from PubChem as 3D SDF conformers when available or generated using ChemDraw. Receptors and ligands were converted into PDBQT format using Open Babel v2.4.1 [117].

Docking simulations were carried out using AutoDock Vina v1.1.2 [118]. Grid boxes were centered on the STAT3 DNA-binding interface, EGFR ATP-binding pocket, PTGS2 cyclooxygenase channel, and AKT1 ATP-binding pocket. Grid center coordinates and box dimensions are provided in Supplementary Table S24. Exhaustiveness was set to 24, with 20 output poses and an energy range of 4 kcal/mol. The best-ranked pose within the predefined binding region was selected for interaction analysis. Poses with docking scores ≤ −7.0 kcal/mol and appropriate binding-site accommodation were selected for detailed 2D and 3D visualization using BIOVIA Discovery Studio Visualizer v21.1.0.20298 [119], following a previously reported workflow [120]. Docking protocol validation was performed by redocking the co-crystallized ligands of EGFR, PTGS2, and AKT1 using the same grid parameters and docking settings. The crystallographic and top-ranked redocked poses were converted to MOL2 format, and the RMSD values between the corresponding poses were calculated using DockRMSD [121]. RMSD values ≤ 2.0 Å were considered indicative of successful pose reproduction [122]. Redocking was not applicable to STAT3 because docking was directed toward the DNA-binding interface rather than a co-crystallized small-molecule binding site.

## 5. Conclusions

This study highlights *Sphagneticola trilobata* as a promising source of antioxidant phenolic- and flavonoid-rich fractions. Among the investigated samples, the flower ethyl acetate fraction showed the strongest activity, exceeding isolated butein and supporting a potential multi-constituent fraction-level effect. The integrated computational workflow prioritized putative metabolites, targets, and pathways potentially associated with redox homeostasis and oxidative-stress responses, including inflammatory, transcriptional, kinase-mediated, and stress-adaptive signaling. These in silico findings should be regarded as predictive hypotheses regarding possible molecular mechanisms rather than established mechanistic evidence and therefore require validation in cellular oxidative stress models and target-specific assays. Future evaluation of the residual hydroethanolic fraction, additional plant organs and extraction solvents, and broader biological activities may further extend the phytochemical and pharmacological characterization of *S. trilobata*.

## Figures and Tables

**Table 1:** Metabolites tentatively identified in the ethyl acetate fractions of *S. trilobata* flowers and leaves using UPLC–ESI–MS/MS in positive and negative ionization modes.

No.	R_t_	Mwt	[M^+^+H]^+^	[M^+^−H]^−^	MS^2^ Fragments	Name	Type	Percent in Flowers	Percent in Leaves	Ref.
1	0.49	180		179		Caffeic acid	Phenolic acid	–	0.29	[59]
2	0.68	434		433	271[M^+^-H-162],191,135[M^+^-H-162-C_7_H_5_O_3_]	Naringenin-7-*O*-glucoside	Flavanone	–	0.49	[60]
3	0.71	516	517	515	515, 353, 317, 299	3,4-Dicaffeoylquinic acid	Hydroxy carboxylic acid	20.35	–	[50,61]
4	0.74	117	118			Valine	Amino acid	–	5.55	[62]
5	0.78	196		195	177[M^+^-H-H_2_O]	Maltonic acid	Organic acid	–	1.27	
6	0.90	516	517	515	515, 435, 353, 335, 317, 299	Dicaffeoylquinic acid isomer	Hydroxy carboxylic acid	12.84	–	[50,61]
7	1.04	272	273	271	253, 161, 135, 134, 91	Butin	Flavanone	5.90	17.02	[56,58]
8	1.11	270	271	269	269, 241, 223, 135, 133	8,3′,4′-Trihydroxy flavone	Flavone	9.38	–	
9	1.17	194		193	178, 161, 134, 133	Ferulic acid	Phenolic acid	–	2.96	[16]
10	1.41	272		271	253, 161, 135, 134, 91	Butein (Butin isomer) *	Chalcone	44.35	–	[55,56]
11	1.44	328		327	270[M^+^-CO-OCH_3_]	4′-Hydroxy-5,6,7-trimethoxyflavone	Flavone	–	2.14	[63]
12	1.74	330		329	299[M^+^-H-OCH_3_]	3,5,8-Trihydroxy -6,7-dimethoxy-flavone	Flavone	–	2.68	[64]
13	4.23	318	319			3,3′,4′,5,5′,7-Hexahydroxyflavone	Flavone	–	4.39	
14	16.82	146		145	101 [M^+^-H-COO]	Coumarin	Coumarin	–	0.65	[24]
15	17.92	256	257		135, 119, 91	7,4′-dihydroxy flavanone	Flavanone	–	10.28	[65]
16	18.86	282	283		284[M^+^+2H], 136	7,4′-Dimethoxy flavone	Flavone	–	29.77	[66]

* Compound isolated in the present study. –, not detected.

**Table 2:** Antioxidant activity of *S. trilobata* fractions and isolated butein expressed as IC_50_ values (µg/mL) in ABTS and FRAP assays.

Sample	IC_50_ (ugμg/mL) ± S.D	
	ABTS	FRAP
Flowers methylene chloride fraction	37.45 ± 1.37	56.6 ± 1.82
Flowers ethyl acetate fraction	5.85 ± 0.14	9.11 ± 0.75
Butein	20.0 ± 1.59	59.96 ± 1.25
Ascorbic acid	10.66 ± 0.63	20.89 ± 1.25
Leaves petroleum ether fraction	161.35 ± 4.92	241.29 ± 6.09
Leaves methylene chloride fraction	45.10 ± 1.84	101.41 ± 3.47
Leaves ethyl acetate fraction	11.45 ± 0.63	14.53 ± 0.92

Values are expressed as mean ± SD. Lower IC_50_ values indicate stronger antioxidant activity.

**Table 3:** Core protein targets prioritized from the PPI networks of STF-EAF and STL-EAF and ranked within each fraction in descending order according to degree centrality. DC, degree centrality; BC, betweenness centrality; CC, closeness centrality.

STF-EAF				STL-EAF			
Target Name	DC	BC	CC	Target Name	DC	BC	CC
STAT3	25	404.561345	0.280612	AKT1	33	987.6082	0.279245
EGFR	22	350.4572453	0.276382	JUN	28	411.8377	0.271062
JUN	21	196.0491945	0.275	STAT3	27	418.5124	0.268116
CTNNB1	21	238.0994959	0.273632	CTNNB1	25	281.0326	0.262411
PTGS2	20	504.0569629	0.268293	EGFR	24	252.5706	0.263345
BCL2	18	102.285495	0.269608	PTGS2	23	702.7187	0.259649
MAPK1	17	186.0517356	0.26699	BCL2	23	148.1817	0.261484
MMP9	16	146.2642239	0.264423	MMP9	22	375.9868	0.260563
TLR4	16	223.0349857	0.258216	ESR1	21	427.1523	0.254296
ESR1	16	350.98261	0.260664	NFKB1	21	106.765	0.258741
RELA	15	96.8154361	0.264423	TLR4	18	362.3637	0.25256
KRAS	14	104.0693806	0.260664	APP	17	247.586	0.244224
				CCND1	17	164.4104	0.246667
				PPARG	16	120.2148	0.255172

**Table 4:** Summary of key predicted ADME and drug-likeness descriptors of the non-redundant metabolites identified in STF-EAF and STL-EAF using SwissADME.

Compound	Fraction(s)	TPSA	Consensus LogP	GI Absorption	BBB Permeant	P-gp Substrate	Lipinski Violations	Bioavailability Score
3,4-Dicaffeoylquinic acid	STF-EAF	211.28	0.67	Low	No	Yes	3	0.11
Butin	STF-EAF; STL-EAF	86.99	1.72	High	No	Yes	0	0.55
8,3′,4′-Trihydroxyflavone	STF-EAF	90.90	1.39	High	No	No	0	0.55
Butein	STF-EAF	97.99	2.03	High	No	No	0	0.55
Caffeic acid	STL-EAF	77.76	0.92	High	No	No	0	0.56
Naringenin-7-*O*-glucoside	STL-EAF	166.14	−0.31	Low	No	No	1	0.55
Valine	STL-EAF	63.32	−1.24	High	No	No	0	0.55
Maltonic acid	STL-EAF	138.45	−3.01	Low	No	No	1	0.56
Ferulic acid	STL-EAF	66.76	1.29	High	Yes	No	0	0.85
4′-Hydroxy-5,6,7-trimethoxyflavone	STL-EAF	78.13	2.01	High	Yes	No	0	0.55
3,5,8-Trihydroxy-6,7-dimethoxyflavone	STL-EAF	109.36	1.49	High	No	No	0	0.55
3,3′,4′,5,5′,7-Hexahydroxyflavone	STL-EAF	151.59	0.38	Low	No	No	1	0.55
Coumarin	STL-EAF	30.21	1.20	High	Yes	No	0	0.55
4′,7-Dihydroxyflavanone	STL-EAF	66.76	2.15	High	Yes	Yes	0	0.55
7,4′-Dimethoxyflavone	STL-EAF	48.67	2.51	High	Yes	No	0	0.55

**Table 5:** Docking scores of STF-EAF and STL-EAF metabolites against selected network-prioritized targets.

Fraction	Ligand	STAT3 (6QHD)	EGFR (5U8L)	PTGS2 (5IKR)	AKT1 (4GV1)
STF-EAF	Butein	−6.9	**−7.8**	**−7.8**	-
	3,4-Dicaffeoylquinic acid	**−7.3**	**−9.0**	−6.3	-
	Butin	−6.8	**−7.7**	**−8.5**	-
	8,3′,4′-Trihydroxyflavone	**−7.0**	**−8.0**	**−8.8**	-
STL-EAF	Ferulic acid	−5.5	-	−6.9	−6.4
	3,5,8-Trihydroxy-6,7-dimethoxyflavone	−6.5	-	−6.6	**−8.0**
	7,4′-Dimethoxyflavone	−6.8	-	**−7.9**	**−7.6**
	Caffeic acid	−5.7	-	−6.6	−6.8
	4′-Hydroxy-5,6,7-trimethoxyflavone	−6.4	-	−6.3	**−7.6**

Values are expressed as docking scores in kcal/mol. Bold values indicate complexes with docking scores ≤ −7.0 kcal/mol. that were selected for detailed 2D/3D interaction visualization. The symbol “-” indicates that the target was not selected for docking with the corresponding fraction.

## Data Availability

The original contributions presented in this study are included in the article/Supplementary Material. Further inquiries can be directed to the corresponding authors.

## Supplementary Materials

The following supporting information can be downloaded at https://www.mdpi.com/article/10.3390/ph19081252/s1, Figure S1: UPLC–ESI–MS/MS chromatograms of the petroleum ether fractions of *S. trilobata* flowers and leaves in positive ionization mode (a, c) and negative ionization mode (b, d), respectively.; Figure S2: Representative chemical structures of metabolites tentatively identified in the petroleum ether fractions of *S. trilobata* flowers and leaves: (a) diterpenes and (b) other metabolite classes.; Figure S3: Representative negative-mode ESI–MS/MS spectra of selected diterpenes tentatively identified in the petroleum ether fractions of *S. trilobata* flowers and leaves.; Figure S4: UPLC–ESI–MS/MS chromatograms of the methylene chloride fractions of *S. trilobata* flowers and leaves in positive ionization mode (a, c) and negative ionization mode (b, d), respectively.; Figure S5: Representative chemical structures of metabolites tentatively identified in the methylene chloride fractions of *S. trilobata* flowers and leaves.; Figure S6: Representative positive- and negative-mode ESI–MS/MS spectra of selected flavonoids tentatively identified in the ethyl acetate fractions of *S. trilobata* flowers and leaves.; Figure S7: IC_50_ bar graphs of *Sphagneticola trilobata* fractions, isolated butein, and ascorbic acid in antioxidant assays. (A) ABTS radical scavenging assay. (B) Ferric reducing antioxidant power assay.; Figure S8: 3D binding poses of selected STF-EAF metabolite–target complexes with docking scores ≤ −7.0 kcal/mol. (A) 3,4-Dicaffeoylquinic acid-STAT3; (B) 8,3′,4′-trihydroxyflavone-STAT3; (C) butein-EGFR; (D) 3,4-dicaffeoylquinic acid-EGFR; (E) butin-EGFR; (F) 8,3′,4′-trihydroxyflavone-EGFR; (G) butein-PTGS2; (H) butin-PTGS2; (I) 8,3′,4′-trihydroxyflavone-PTGS2. The corresponding 2D interaction maps are shown in Figure 10. STF-EAF, *S. trilobata* flower ethyl acetate fraction.; Figure S9: 3D binding poses of selected STL-EAF metabolite–target complexes with docking scores ≤ −7.0 kcal/mol. (A) 3,5,8-Trihydroxy-6,7-dimethoxyflavone-AKT1; (B) 7,4′-dimethoxyflavone-AKT1; (C) 4′-hydroxy-5,6,7-trimethoxyflavone-AKT1; (D) 7,4′-dimethoxyflavone-PTGS2. The corresponding 2D interaction maps are shown in Figure 11. STL-EAF, *S. trilobata* leaf ethyl acetate fraction.; Table S1: Tentatively identified metabolites in the petroleum ether fractions of *S. trilobata* flowers and leaves using UPLC–ESI–MS/MS in positive and negative ionization modes.; Table S2: Metabolites tentatively identified in the methylene chloride fractions of *S. trilobata* flowers and leaves using UPLC–ESI–MS/MS in positive and negative ionization modes.; Table S3: Non-redundant predicted targets of STF-EAF metabolites generated by SwissTargetPrediction after standardization to official gene symbols and removal of duplicate entries.; Table S4: Non-redundant predicted targets of STL-EAF metabolites generated by SwissTargetPrediction after standardization to official gene symbols and removal of duplicate entries.; Table S5: Oxidative stress-related genes retrieved from GeneCards and OMIM, standardized to official gene symbols, merged, and deduplicated to generate the final oxidative stress-related target set.; Table S6: Putative antioxidant-relevant overlapping targets identified by intersecting STF-EAF predicted targets with oxidative stress-related genes.; Table S7: Putative antioxidant-relevant overlapping targets identified by intersecting STL-EAF predicted targets with oxidative stress-related genes.; Table S8: Topological parameters of the initial STF-EAF PPI network constructed using STRING after hiding disconnected nodes.; Table S9: Topological parameters of the STF-EAF primary PPI subnetwork retained after the first degree-based filtering step using DC ≥ 6.; Table S10: Topological parameters of the initial STL-EAF PPI network constructed using STRING after hiding disconnected nodes.; Table S11: Topological parameters of the STL-EAF primary PPI subnetwork retained after the first degree-based filtering step using DC ≥ 6.; Table S12: Degree-based ranking of STF-EAF metabolites according to their predicted connectivity with antioxidant-relevant overlapping targets in the compound–target network.; Table S13: Degree-based ranking of STL-EAF metabolites according to their predicted connectivity with antioxidant-relevant overlapping targets in the compound–target network.; Table S14: Significantly enriched GO biological process terms associated with STF-EAF core hub targets, identified using DAVID with Benjamini-adjusted *p*-value ≤0.05.; Table S15: Significantly enriched GO cellular component terms associated with STF-EAF core hub targets, identified using DAVID with Benjamini-adjusted *p*-value ≤0.05.; Table S16: Significantly enriched GO molecular function terms associated with STF-EAF core hub targets, identified using DAVID with Benjamini-adjusted *p*-value ≤0.05.; Table S17: Significantly enriched KEGG pathways associated with STF-EAF core hub targets, identified using DAVID with Benjamini-adjusted *p*-value ≤0.05.; Table S18: Significantly enriched GO biological process terms associated with STL-EAF core hub targets, identified using DAVID with Benjamini-adjusted *p*-value ≤0.05.; Table S19: Significantly enriched GO cellular component terms associated with STL-EAF core hub targets, identified using DAVID with Benjamini-adjusted *p*-value ≤0.05.; Table S20: Significantly enriched GO molecular function terms associated with STL-EAF core hub targets, identified using DAVID with Benjamini-adjusted *p*-value ≤0.05.; Table S21: Significantly enriched KEGG pathways associated with STL-EAF core hub targets, identified using DAVID with Benjamini-adjusted *p*-value ≤0.05.; Table S22: Complete SwissADME-predicted ADME and drug-likeness properties of the non-redundant metabolites identified in STF-EAF and STL-EAF.; Table S23: Detailed interaction parameters of selected metabolite–target complexes visualized in 2D and 3D docking maps.; Table S24: Grid box parameters and native-ligand redocking results for the selected protein targets.

## Abbreviations

The following abbreviations are used in this manuscript:

2D	Two-dimensional
3D	Three-dimensional
^13^C-NMR	Carbon-13 nuclear magnetic resonance
^1^H-NMR	Proton nuclear magnetic resonance
ABTS	2,2′-Azinobis(3-ethylbenzothiazoline-6-sulphonic acid)
ADME	Absorption, distribution, metabolism, and excretion
AGE-RAGE	Advanced glycation end products-receptor for advanced glycation end products
AIDS	Acquired immunodeficiency syndrome
AKT1	AKT serine/threonine kinase 1
AlCl_3_	Aluminum chloride
ANOVA	Analysis of variance
AP-1	Activator protein 1
APP	Amyloid beta precursor protein
ATP	Adenosine triphosphate
BBB	Blood–brain barrier
BC	Betweenness centrality
BCL2	B-cell lymphoma 2
BEH	Ethylene-bridged hybrid
BHA	Butylated hydroxyanisole
BHT	Butylated hydroxytoluene
BP	Biological process
CC (GO)	Cellular component
CC (network topology)	Closeness centrality
CCND1	Cyclin D1
CD_3_OD	Deuterated methanol
CH_2_Cl_2_	Dichloromethane
COX2	Cyclooxygenase-2
CTNNB1	Catenin beta 1
DAVID	Database for Annotation, Visualization and Integrated Discovery
DC	Degree centrality
DNA	Deoxyribonucleic acid
DPPH	2,2-Diphenyl-1-picrylhydrazyl
EGFR	Epidermal growth factor receptor
ESI	Electrospray ionization
ESI-MS/MS	Electrospray ionization tandem mass spectrometry
ESR1	Estrogen receptor 1
FRAP	Ferric reducing antioxidant power
GI	Gastrointestinal
GO	Gene Ontology
H_3_BO_3_	Boric acid
HCl	Hydrochloric acid
HIF-1	Hypoxia-inducible factor 1
HMBC	Heteronuclear multiple bond correlation
HPLC	High-performance liquid chromatography
HSQC	Heteronuclear single quantum coherence
IC_50_	Half-maximal inhibitory concentration
IL-17	Interleukin-17
JUN	Jun proto-oncogene, AP-1 transcription factor subunit
KEGG	Kyoto Encyclopedia of Genes and Genomes
KRAS	KRAS proto-oncogene, GTPase
LC-MS/MS	Liquid chromatography-tandem mass spectrometry
MAPK	Mitogen-activated protein kinase
MAPK1	Mitogen-activated protein kinase 1
MeOH	Methanol
MF	Molecular function
MMP9	Matrix metalloproteinase 9
MRM	Multiple-reaction monitoring
MS	Mass spectrometry
MS/MS	Tandem mass spectrometry
Mwt	Molecular weight
m/z	Mass-to-charge ratio
NaOAc	Sodium acetate
NaOMe	Sodium methoxide
NDGA	Nordihydroguaiaretic acid
NF-κB	Nuclear factor kappa B
NFKB1	Nuclear factor kappa B subunit 1
NIGMS	National Institute of General Medical Sciences
NMR	Nuclear magnetic resonance
OMIM	Online Mendelian Inheritance in Man
PDB	Protein Data Bank
PDBQT	Protein Data Bank, partial charge, and AutoDock atom type format
P-gp	P-glycoprotein
PPI	Protein–protein interaction
PPARG	Peroxisome proliferator-activated receptor gamma
PTGS2	Prostaglandin-endoperoxide synthase 2
RCMB	Regional Center for Mycology and Biotechnology
RCSB	Research Collaboratory for Structural Bioinformatics
RELA	RELA proto-oncogene, NF-κB subunit
Rf	Retention factor
ROS	Reactive oxygen species
Rt	Retention time
*S. trilobata*	*Sphagneticola trilobata*
SD	Standard deviation
SDF	Structure Data File
SMILES	Simplified Molecular Input Line Entry System
STAT3	Signal transducer and activator of transcription 3
STF-EAF	*Sphagneticola trilobata* flower ethyl acetate fraction
STL-EAF	*Sphagneticola trilobata* leaf ethyl acetate fraction
STRING	Search Tool for the Retrieval of Interacting Genes/Proteins
TLC	Thin-layer chromatography
TLR4	Toll-like receptor 4
TNF	Tumor necrosis factor
TPSA	Topological polar surface area
TQD	Tandem quadrupole detector
UPLC	Ultra-performance liquid chromatography
UPLC–ESI–MS/MS	Ultra-performance liquid chromatography coupled with electrospray ionization tandem mass spectrometry
UV	Ultraviolet
UV-A	Ultraviolet A
*v*/*v*	Volume per volume
*W. trilobata*	*Wedelia trilobata*
*w*/*v*	Weight per volume
