# Supplementary material for: Chemical Profiling and Redox-Target Mapping of Antioxidant Fractions from Sphagneticola trilobata: An Integrated UPLC–ESI–MS/MS, Network Pharmacology, and Molecular Docking Study
Source: Pharmaceuticals (Basel). 2026 Aug 9;19(8):1252. doi: 10.3390/ph19081252 (PMC13516535; doi:10.3390/ph19081252)
Supplement: Supplementary file 1 [file pharmaceuticals-19-01252-s001.zip › Supplementary figures S1-S9.pdf]

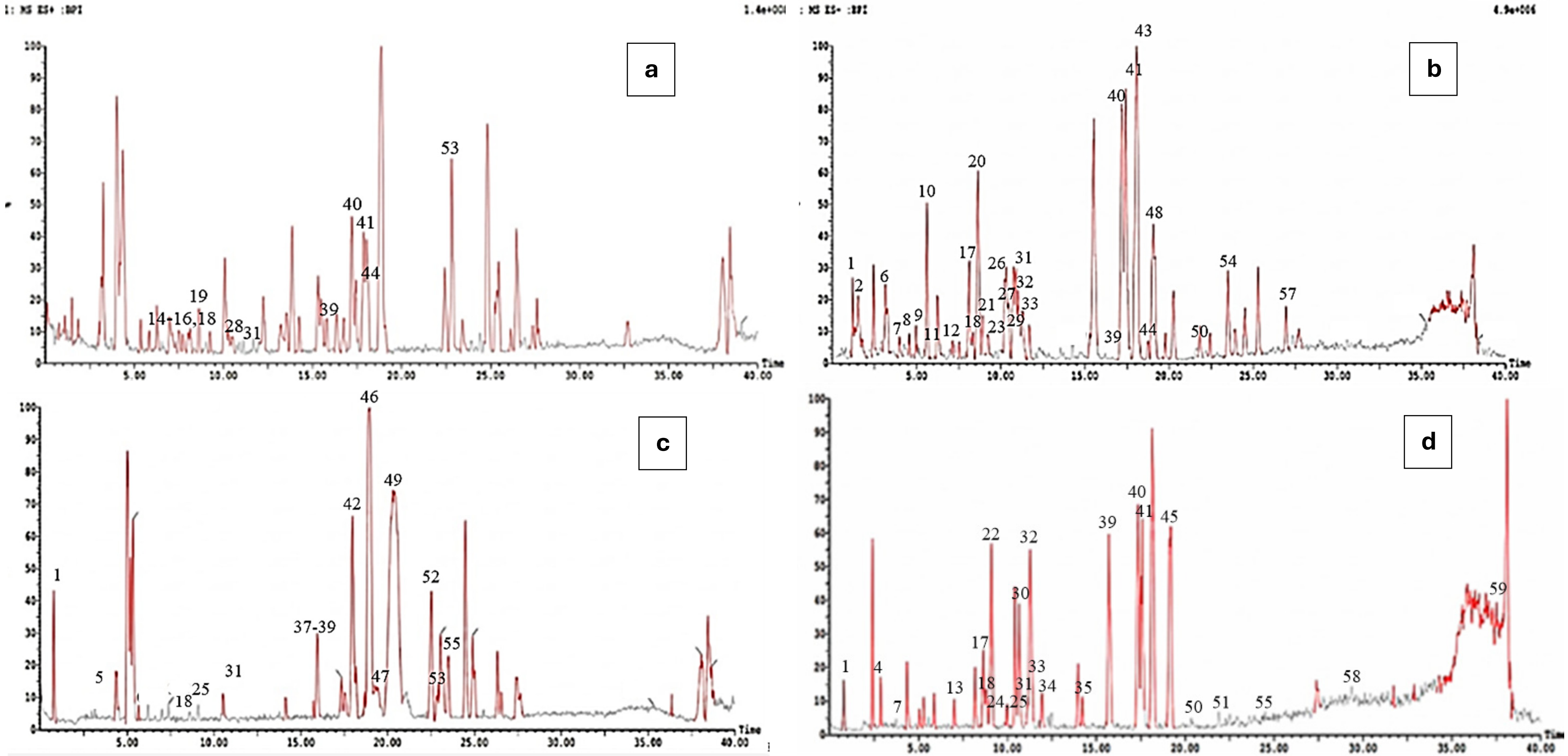


**Supplementary Figure S1.** UPLC–ESI–MS/MS chromatograms of the petroleum ether fractions of *Sphagneticola trilobata* flowers and leaves in positive ionization mode (a, c) and negative ionization mode (b, d), respectively.

**(a)**

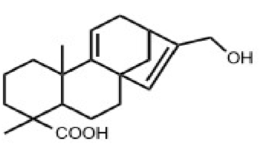

Ent-17-hydroxykaura-9(11),15-dien-19-oic acid (3)

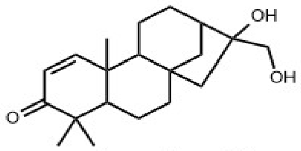

Anomaluone (8)

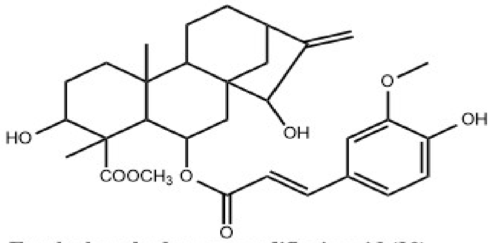

Feruloyloxy hydroxy grandifloric acid (10)

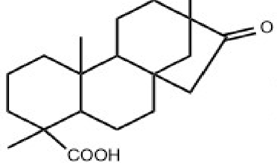

Isosteviol (11)

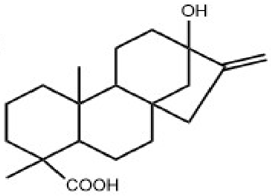

Steviol (12)

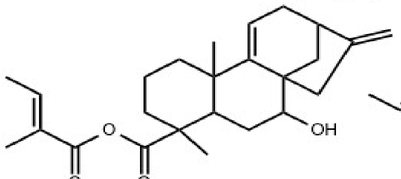

Angeloyloxy grandiflorenic acid (16)

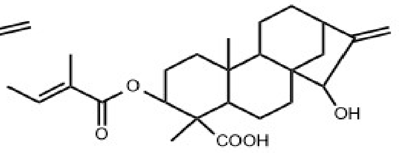

Tigloyloxy grandifloric acid (17)

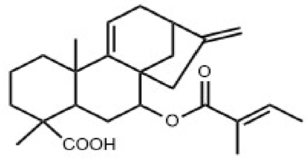

Angeloyloxy grandiflorenic acid isomer (19)

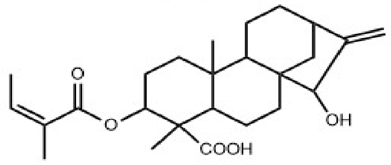

Angeloyloxy grandifloric acid (20)

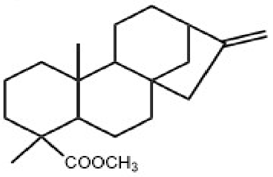

Ent-kaurenoic acid methyl ester (21)

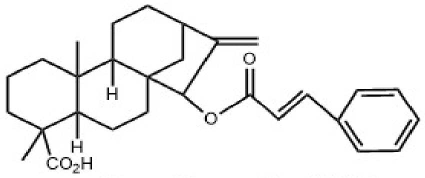

coumaroyloxy entkaurenoic acid (25)

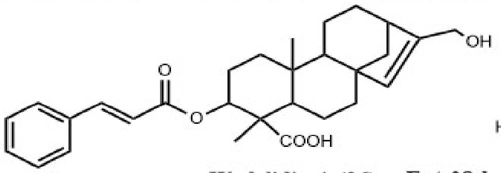

Wedelidin A (26)

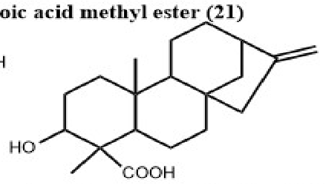

Ent-3ß-hydroxy-kaur-16-en-19-oic acid (29)

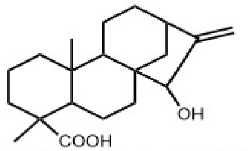

Grandifloric acid (31)

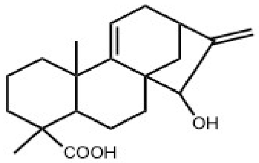

Hydroxy grandiflorenic acid (33)

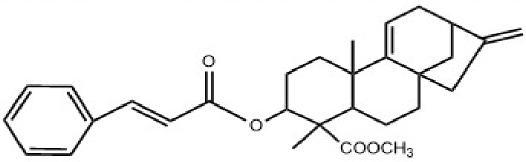

Cinnamoyloxy grandiflorenic acid methyl ester (34)

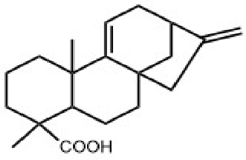

Grandiflorenic acid (39)

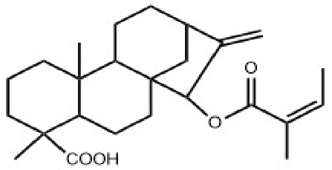

Angeloyloxy entkaurenoic acid (40)

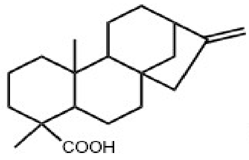

Ent-kaurenoic acid (41)

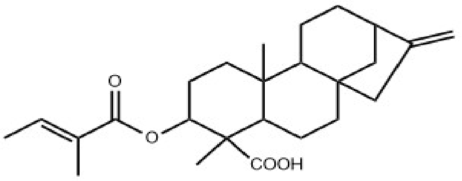

Tigloyloxy ent-kaurenoic acid (43)

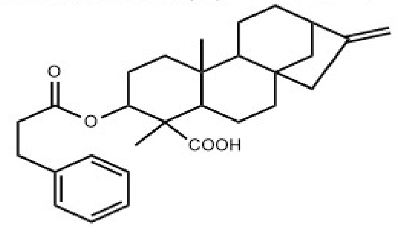

Dihydrocinnamoyloxy ent- kaurenoic acid (44)

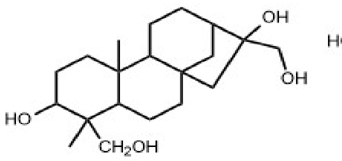

ent-Kaurane-3b,16b,17,19-tertol (49)

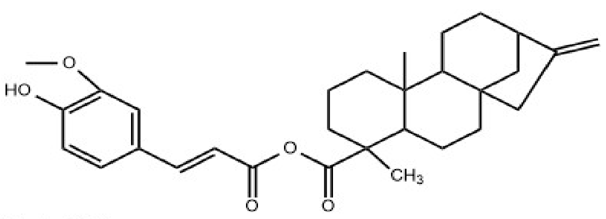

Feruloyloxy ent-kaurenoic acid (57)

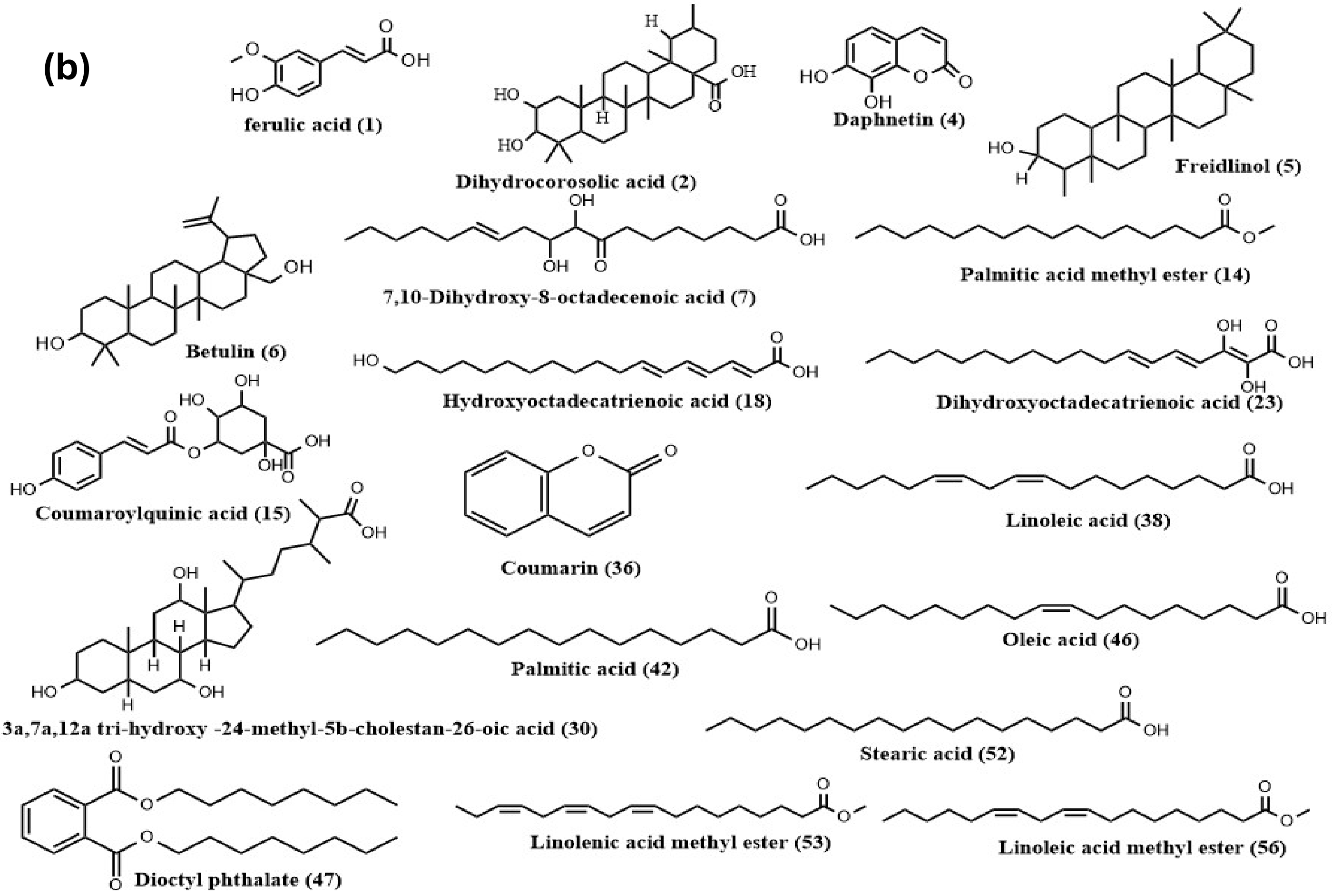


**Supplementary Figure S2.** Representative chemical structures of metabolites tentatively identified in the petroleum ether fractions of *S. trilobata* flowers and leaves: (a) diterpenes and (b) other metabolite classes.

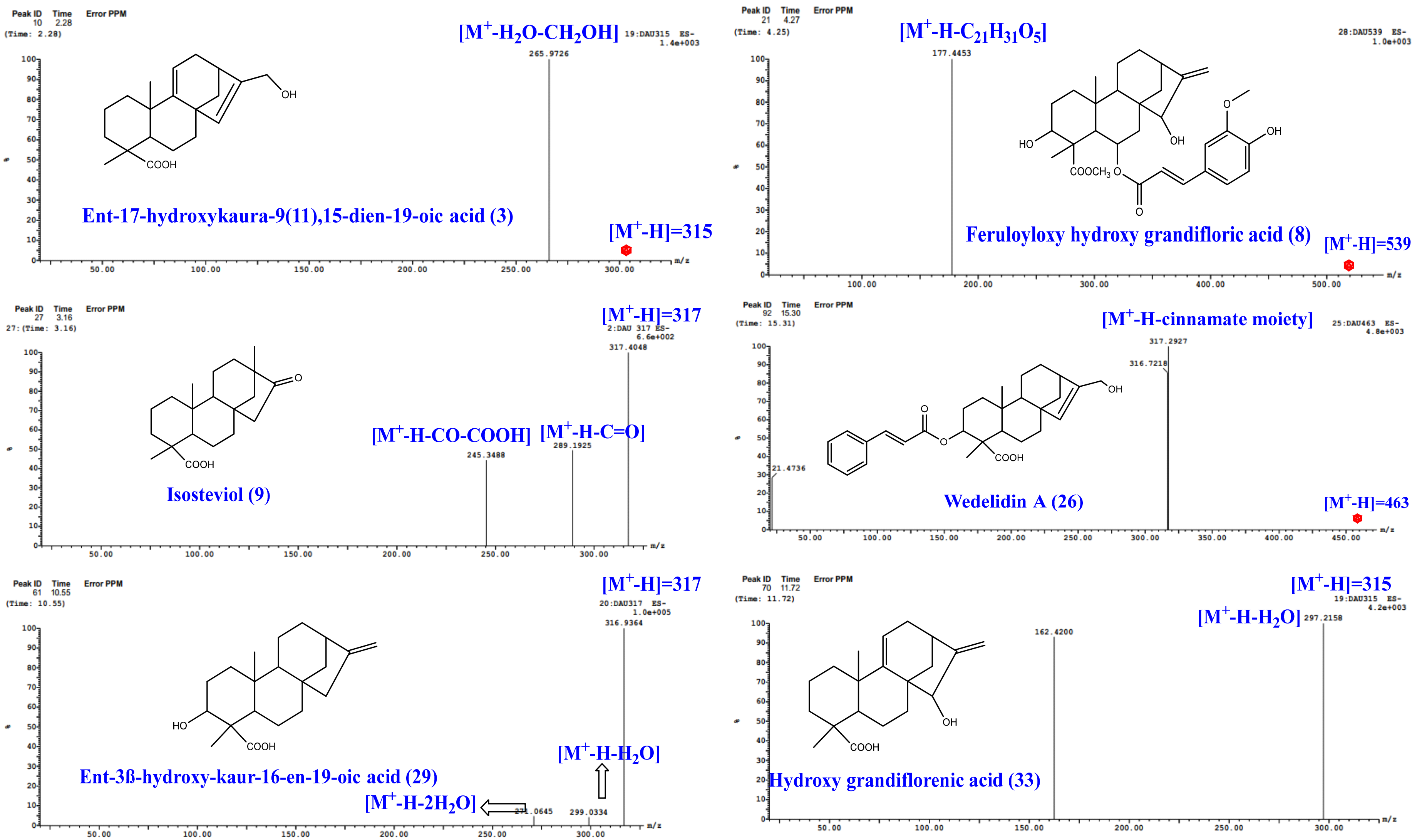

Peak ID Time Error PPM
10 2.28
(Time: 2.28)
[M+-H2O-CH2OH]
19:DAU315 ES-
1.4e+003
265.9726
Ent-17-hydroxykaura-9(11),15-dien-19-oic acid (3)
[M+-H]=315
Peak ID Time Error PPM
21 4.27
(Time: 4.25)
[M+-H-C21H31O5]
28:DAU539 ES-
1.0e+003
177.4453
Feruloyloxy hydroxy grandifloric acid (8)
[M+-H]=539
Peak ID Time Error PPM
27 3.16
27: (Time: 3.16)
[M+-H]=317
2:DAU 317 ES-
6.6e+002
317.4048
[M+-H-CO-COOH]
245.3488
[M+-H-C=O]
289.1925
Isosteviol (9)
Peak ID Time Error PPM
92 15.30
(Time: 15.31)
[M+-H-cinnamate moiety]
25:DAU463 ES-
4.8e+003
317.2927
316.7218
21.4736
Wedelidin A (26)
[M+-H]=463
Peak ID Time Error PPM
61 10.55
(Time: 10.55)
[M+-H]=317
20:DAU317 ES-
1.0e+005
316.9364
Ent-3ß-hydroxy-kaur-16-en-19-oic acid (29)
[M+-H-H2O]
[M+-H-2H2O]
271.0645
299.0334
Peak ID Time Error PPM
70 11.72
(Time: 11.72)
[M+-H]=315
19:DAU315 ES-
4.2e+003
[M+-H-H2O]
297.2158
162.4200
Hydroxy grandiflorenic acid (33)
m/z

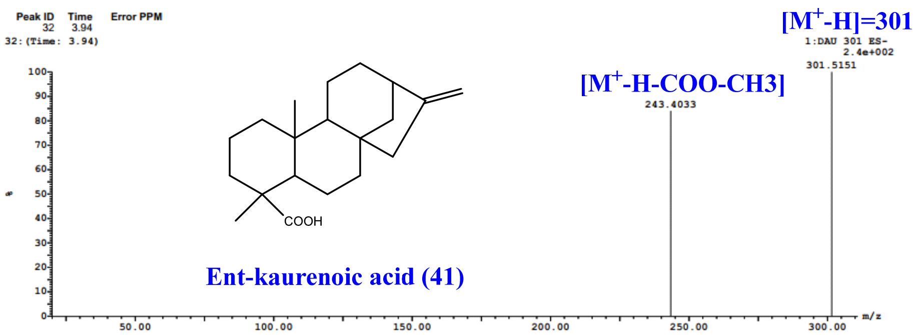


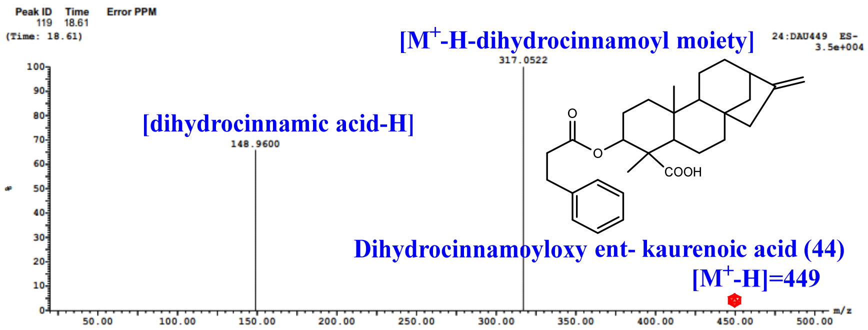


**Supplementary Figure S3.** Representative negative-mode ESI–MS/MS spectra of selected diterpenes tentatively identified in the petroleum ether fractions of *Sphagneticola trilobata* flowers and leaves.

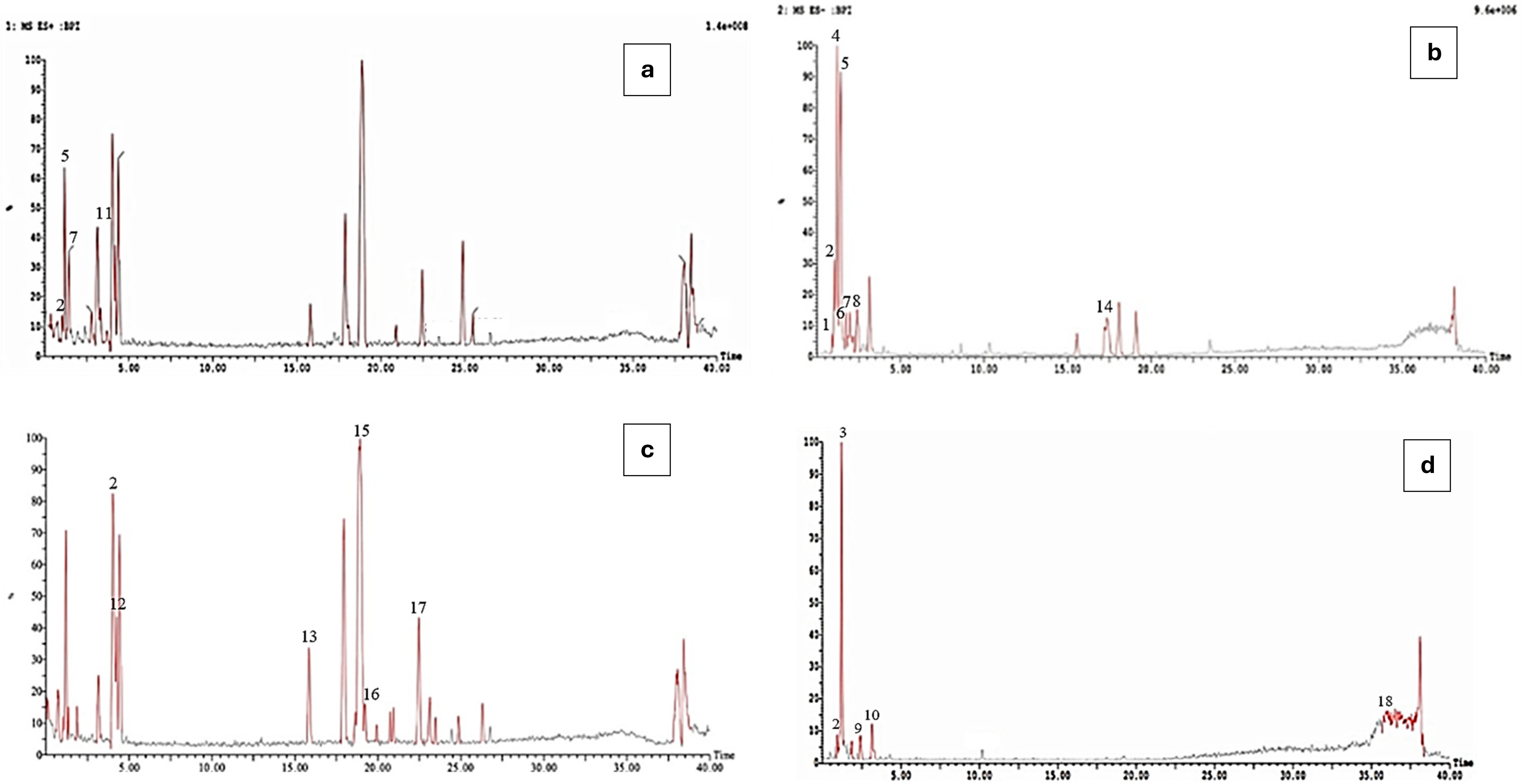


**Supplementary Figure S4.** UPLC–ESI–MS/MS chromatograms of the methylene chloride fractions of *Sphagneticola trilobata* flowers and leaves in positive ionization mode (a, c) and negative ionization mode (b, d), respectively.

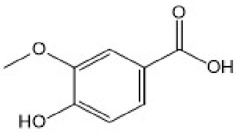


**Vanillic acid (1)**

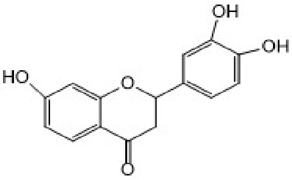


**Butin (2)**

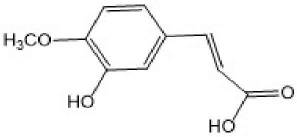


**Isoferulic acid (4)**

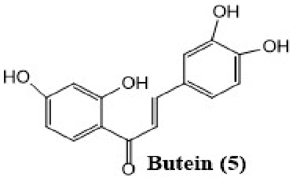


**Butein (5)**

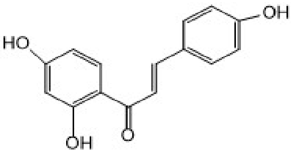


**Liquiritigenin (7,4-dihydroxy flavanone)(6)**

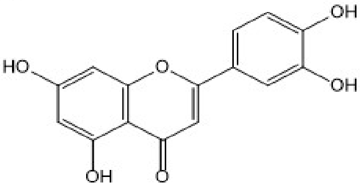


**Luteolin (7)**

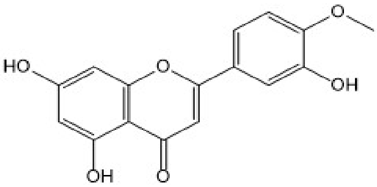


**Diosmetin (8)**

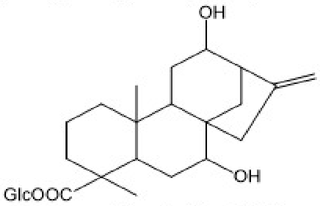


**Naueloside G (10)**

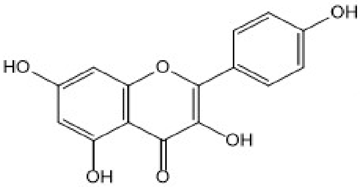


**Kaempferol (11)**

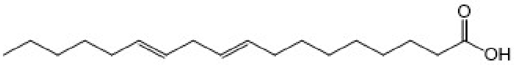


**Linolelaidic acid (13)**

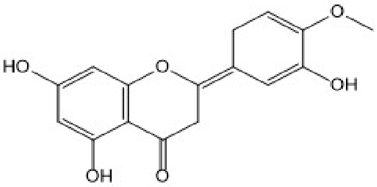


**Hesperetin (14)**

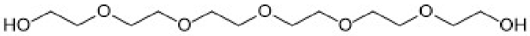


**Hexaethylene glycol (15)**

**Supplementary Figure S5.** Representative chemical structures of metabolites tentatively identified in the methylene chloride fractions of *Sphagneticola trilobata* flowers and leaves.

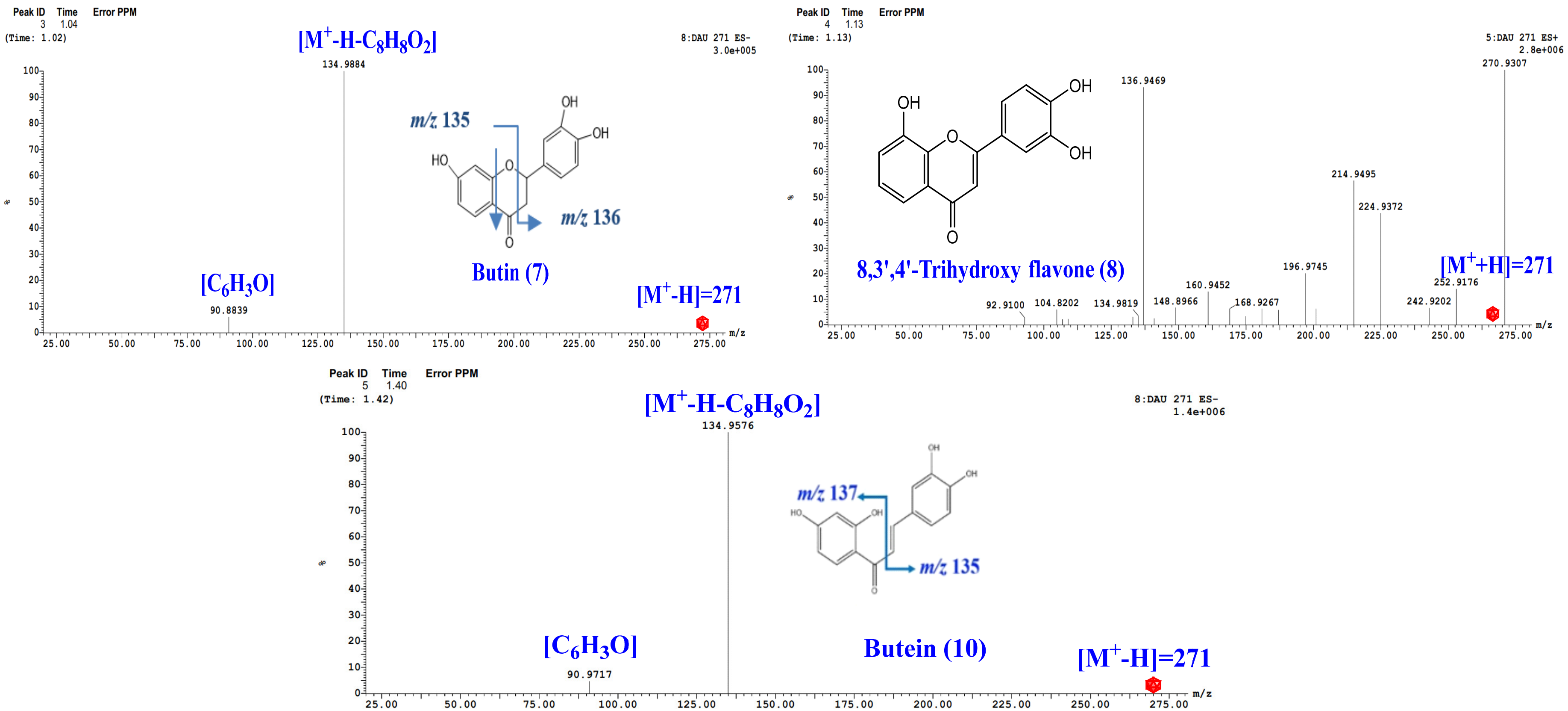


**Supplementary Figure S6.** Representative positive- and negative-mode ESI–MS/MS spectra of selected flavonoids tentatively identified in the ethyl acetate fractions of *Sphagneticola trilobata* flowers and leaves.

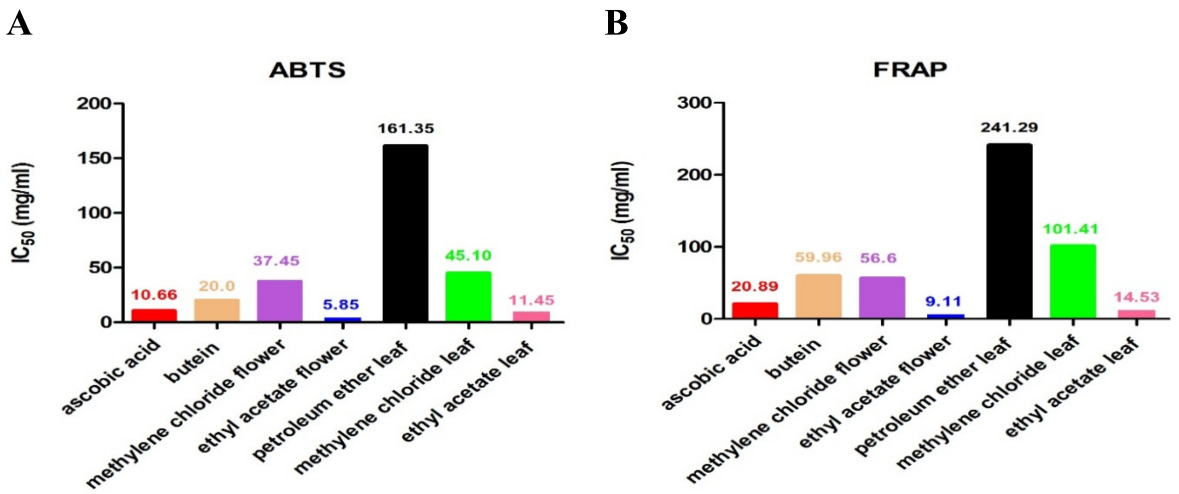


**Supplementary Figure S7.** $IC_{50}$ bar graphs of *Sphagneticola trilobata* fractions, isolated butein, and ascorbic acid in antioxidant assays. (**A**) ABTS radical scavenging assay. (**B**) Ferric reducing antioxidant power assay.

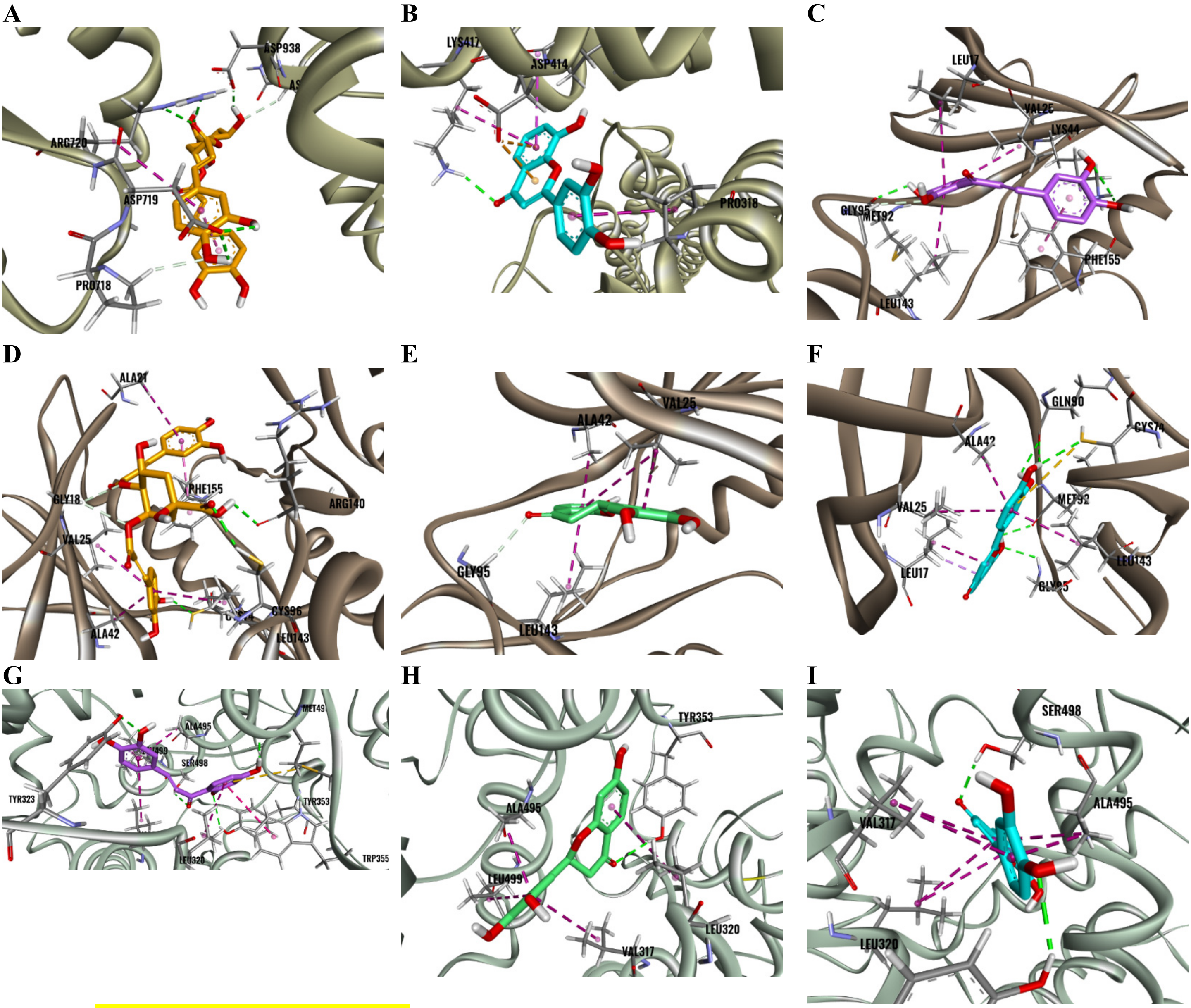


**Supplementary Figure S8.** 3D binding poses of selected STF-EAF metabolite-target complexes with docking scores ≤ −7.0 kcal/mol. (**A**) 3,4-Dicaffeoylquinic acid-STAT3; (**B**) 8,3′,4′-trihydroxyflavone-STAT3; (**C**) butein-EGFR; (**D**) 3,4-dicaffeoylquinic acid-EGFR; (**E**) butin-EGFR; (**F**) 8,3′,4′-trihydroxyflavone-EGFR; (**G**) butein-PTGS2; (**H**) butin-PTGS2; (**I**) 8,3′,4′-trihydroxyflavone-PTGS2. The corresponding 2D interaction maps are shown in Figure 10. STF-EAF, *Sphagneticola trilobata* flower ethyl acetate fraction.

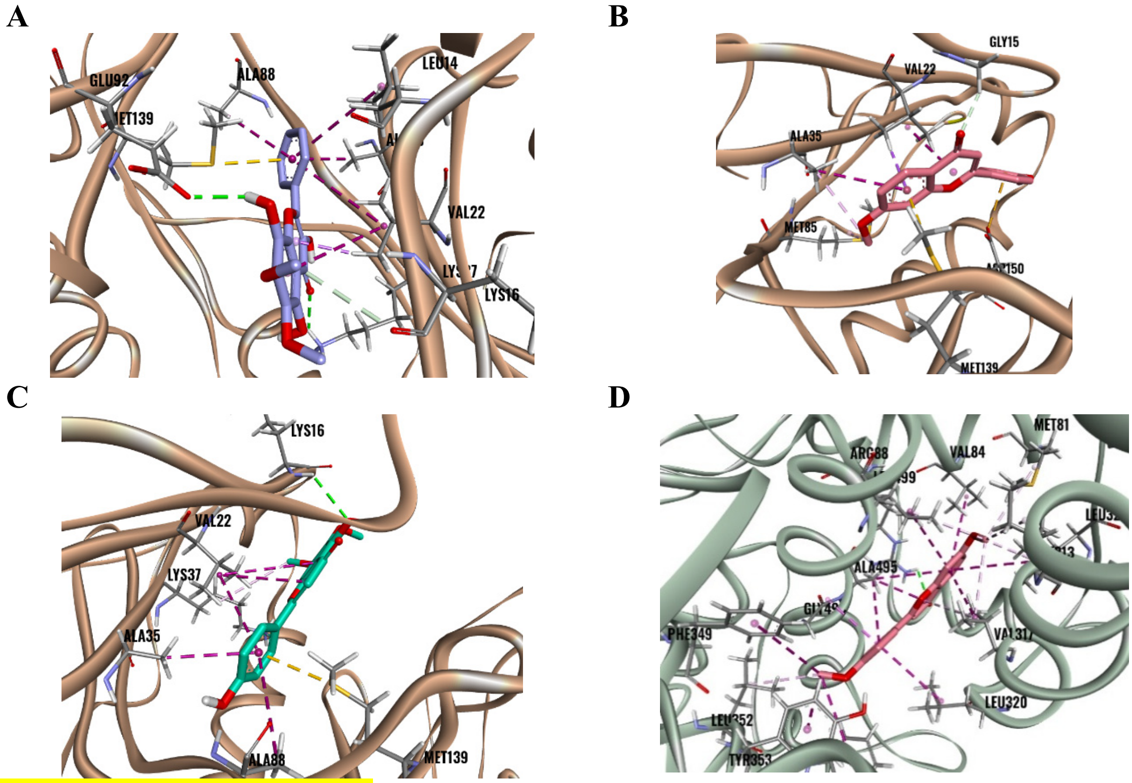


**Supplementary Figure S9.** 3D binding poses of selected STL-EAF metabolite-target complexes with docking scores ≤ −7.0 kcal/mol. (**A**) 3,5,8-Trihydroxy-6,7-dimethoxyflavone-AKT1; (**B**) 7,4′-dimethoxyflavone-AKT1; (**C**) 4′-hydroxy-5,6,7-trimethoxyflavone-AKT1; (**D**) 7,4′-dimethoxyflavone-PTGS2. The corresponding 2D interaction maps are shown in Figure 11. STL-EAF, *S. trilobata* leaf ethyl acetate fraction.
