# Supplementary material for: Chemical Profiling and Redox-Target Mapping of Antioxidant Fractions from Sphagneticola trilobata: An Integrated UPLC–ESI–MS/MS, Network Pharmacology, and Molecular Docking Study
Source: Pharmaceuticals (Basel). 2026 Aug 9;19(8):1252. doi: 10.3390/ph19081252 (PMC13516535; doi:10.3390/ph19081252)
Supplement: Supplementary file 1 [file pharmaceuticals-19-01252-s001.zip › Supplementary tables S1-S2 and S22-24.pdf]

Supplementary Table S1. Tentatively identified metabolites in the petroleum ether fractions of *Sphagneticola trilobata* flowers and leaves using UPLC–ESI–MS/MS in positive and negative ionization modes.

No.	R_t	Mwt	$[M^{+}+H]^{+}$	$[M^{+}-H]^{-}$	MS^2 Fragments	Name	Type	Percent in flower	Percent in leaf	Ref.
1	1.20	194	195	193	178, 161, 134, 133	Ferulic acid	Phenolic acid	1.06	2.40	**[16]**
2	1.75	474		473	427 $[M^{+}-H-COOH]$, 409 $[M^{+}-H-COOH-H_2O]$	Dihydro-corosolic acid	Triterpene	0.49	-	**[43]**
3	2.28	316		315	315, 265 $[M^{+}-H_2O-CH_2OH]$	Ent-17-hydroxykaura-9(11),15-dien-19-oic acid	Diterpene	+	-	**[44]**
4	2.86	178		177	162,145,118,117	Daphnetin	Hydroxy coumarin		0.50	**[42]**
5	3.15	428	429			Freidlinol	Triterpene		1.06	**[6]**
6	3.22	442		441	409$[M^{+}-H-CH_2OH]$	Betulin	Triterpene	0.65	-	**[40,41]**
7	3.98	314		313	313, 295, 267, 195, 183	7,10-Dihydroxy-8-octadecenoic acid	Fatty acid	0.37	0.27	**[39]**
8	4.57	318		317	287 $[M^{+}-H-CH_2OH]$	Anomaluone	Diterpene	0.32	-	**[38]**
9	4.95	316		315	233, 268	Ent-17-hydroxykaura-9(11),15-dien-19-oic acid isomer	Diterpene	0.39	-	**[44]**
10	5.61	540		539	177 $[M^{+}-H-C_{21}H_{31}O_5]^{-}$	Feruloyloxy hydroxy grandifloric acid	Diterpene	2.87	-	**[36,37]**
11	6.23	318		317	289 $[M^{+}-H-C{=}O]$, 245 $[M^{+}-H-CO-COOH]^{-}$	Isosteviol	Diterpene	0.33	-	**[35]**
12	6.25	318		317	271$[M^{+}-H-COOH]$	Steviol	Diterpene	+	-	**[35]**
13	7.85	538		537	179$[M^{+}-H-358]$	Caffeic acid derivative	Phenolic acid	-	0.40	
14	8.01	270	271		271, 239 $[M^{+}+H-32]$	Palmitic acid methyl ester	Fatty acid methyl ester	0.40	-	**[34]**
15	8.01	338	339		339, 147, 91	Coumaroylquinic acid	Hydroxy carboxylic acid	0.40	-	**[33]**
16	8.12	398	399		399, 299, 271 [-CO]	Angeloyloxy grandiflorenic acid	Diterpene	0.50	-	**[44]**

17	8.12	416		415	415, 333 [M^+-H-82 (tigloyl moiety)], 99 (tigloyl)	Tigloyloxy grandifloric acid	Diterpene	2.29	1.27	**[44]**
18	8.35	294	295	293	275 [M^+-H-18], 245, 221, 183, 171	Hydroxyoctadecatrienoic acid	Fatty acid	0.64	0.36, 0.73	**[32]**
19	8.62	398	399		399, 299 [angelic acid], 277 [-H_2O]	Angeloyloxy grandiflorenic acid isomer	Diterpene	0.93	-	**[44]**
20	8.62	416		415	415, 99	Angeloyloxy grandifloric acid	Diterpene	4.61	-	**[44]**
21	8.86	316		315	----	Ent-kaurenoic acid methyl ester	Diterpene	+	-	**[30,31]**
22	9.10	416		415	415[M^+-H],99[M^+-H-grandifloric acid]	Tigloyloxy grandifloric acid isomer	Diterpene	-	3.60	**[44]**
23	9.23	310		309	291[M^+-H-H_2O], 263	Dihydroxy-octadecatrienoic acid	Fatty acid	+	-	**[32]**
24	9.25	538		537	138[M^+-H-399]	Hydroxy benzoic acid derivative	Carboxylic acid	-	0.40	
25	9.98	448		447	147[Bp M^+-H-grandifloric acid],102[147-COOH]	Cinnamoyloxy grandifloric acid or coumaroyloxy entkaurenoic acid	Diterpene	-	0.31, 5.71	
26	10.33	464		463	317, 316	Wedelidin A	Diterpene	3.85	-	**[6,29]**
27	10.42	560		559	455[M^+-H-104]	Betulinic acid derivative	Triterpene	1.93		**[41]**
28	10.48	298	299		299, 253	Methylated stearic acid	Fatty acid methyl ester	0.59	-	**[34]**
29	10.55	318		317	317, 299, 271	Ent-3β-hydroxy-kaur-16-en-19-oic acid	Diterpene	+	-	**[28,45]**
30	10.64	464		463	463,445,417,362,209,149,147	3α,7α,12α trihydroxy -24-methyl-5β-cholestan-26-oic acid		-	1.89	**[27]**
31	10.75	318	319	317	299 [M^+-H-H_2O], 271 [M^+-H-COOH]	Grandifloric acid	Diterpene	2.03	0.40 and 0.32	**[26,44]**

32	11.27	556		555	302[M^{+}-H-253]	Entkaurenoic acid derivative	Diterpene	1.92	4.18, 1.93	**[6]**
33	11.72	316		315	297 [M^{+}-H-H$_2$O]	Hydroxy grandiflorenic acid	Diterpene	+	0.51	**[25,44]**
34	11.94	462		461	147[M^{+}-H-314]	Cinnamoyloxy grandiflorenic acid methyl ester	Diterpene		0.62	
35	14.22	538		537	148[M^{+}-H-389]	Cinnamic acid derivative	Carboxylic acid		0.40	
36	14.51	146		145	101[M^{+}-H-COO]	Coumarin	Coumarin		11.78	**[24]**
37	15.74	300	301		255[M^{+}+H-COOH]	Grandiflorenic acid isomer	Diterpene		0.18	**[26]**
38	15.94	280	281		281[M^{+}+H],280[M^{+}+H-H],263[M^{+}+H-H$_2$O]	Linoleic acid	Fatty acid		2.14	**[23]**
39	16.89	300	301	299	301, 283 [M^{+}+H- H$_2$O], 255 [M^{+}+H- H$_2$O -CO], 199	Grandiflorenic acid	Diterpene	+	4.27	**[3,9]**
40	17.21	400	401	399	399, 398, 317, 99	Angeloyloxy ent-kaurenoic acid	Diterpene	7.93	4.07, 7.71	**[45]**
41	17.37	302	303	301	243 [M^{+}-H-COO-CH$_3$]$^{-}$	Ent-kaurenoic acid	Diterpene	6.69	2.31	**[18,29]**
42	17.98	256	257			Palmitic acid	Fatty acid		6.66	**[22]**
43	18.5	400		399	317, 99	Tigloyloxy ent-kaurenoic acid	Diterpene	11.89		**[29]**
44	18.61	450		449	317, 149	Dihydrocinnamoyloxy ent- kaurenoic acid isomer	Diterpene	0.39		**[21]**
45	18.73	560		559	313[M^{+}-H-246]	Grandiflorenic acid methyl eater derivative	Diterpene		1.93	
46	18.95	282	283		283[M^{+}+H],282[M^{+}+H-H],265[M^{+}+H-H$_2$O]	Oleic acid	Fatty acid		16.62	
47	19.28	390	391		413[M^{+}+Na],391[M^{+}+H], 279[M^{+}-C$_8$H$_{17}$], 148[M^{+}+H-C$_8$H$_{17}$O-C$_8$H$_{17}$]	Dioctyl phthalate			0.87, 0.68, 0.54	**[20]**

48	19.79	450		449	317,149	Dihydrocinnamoyloxy ent- kaurenoic acid isomer	Diterpene	5.31		**[21]**
49	20.28	338	339			ent-Kaurane-3b,16b,17,19-tertol	Diterpene		25.97	**[19]**
50	21.91	474		473	299	Grandiflorenic acid derivative	Diterpene	+	1.93	**[3,9]**
51	22.11	462		461	99[M^+-H-362]	Anglyloxy diterpene or angelic acid derivative	Diterpene		0.62	
52	22.51	284	285			Stearic acid	Fatty acid		4.18	
53	22.82	292	293		293, 261 [M^++H-32]	Linolenic acid methyl ester	Fatty acid methyl ester	4.78	0.41 and 0.46	**[34]**
54	23.50	450		449	317, 149 (dihydrocinnamoyl moiety)	Dihydrocinnamoyloxy ent- kaurenoic acid	Diterpene	2.10		**[21]**
55	23.87	556		555	362[M^+-H-193]	Ferulic acid derivative	Phenolic acid		4.18	**[16]**
56	25.25	294	295		295 $[M^++H]^+$, 263 $[M^++H-32]^+$	Linoleic acid methyl ester	Fatty acid methyl ester	0.64		**[34]**
57	26.96	478		477	301 $[M^+-H-176]^-$	Feruloyloxy ent-kaurenoic acid	Diterpene	0.90		**[18,46]**
58	29.16	556		555	122[M^+-H-433]	Benzoic acid derivative	Benzoic acid		4.18	
59	37.27	412		411	411,313	Stigmasterol	Sterol		2.50	**[17]**

+= present as minor constituent. LC–MS/MS-only assignments represent tentative candidate structures. Distinct retention times and fragmentation patterns were used to distinguish chromatographic features, but exact positional and stereochemical identities require confirmation using authentic standards or complementary spectroscopic methods.

Supplementary Table S2. Metabolites tentatively identified in the methylene chloride fractions of *Sphagneticola trilobata* flowers and leaves using UPLC–ESI–MS/MS in positive and negative ionization modes.

No.	R_t	Mwt	$[M^{+}+H]^{+}$	$[M^{+}-H]^{-}$	MS^2 Fragments	Name	Type	Percent in flower	Percent in leaf	Ref.
1	1.02	168		167	152, 107	Vanillic acid	Phenolic acid	0.62		**[8,44]**
2	1.02	272	273	271	253, 161, 135, 134, 91	Butin	Flavanone	5.13	12.80	**[56,58]**
3	1.17	194		193	178,161,134,133	Ferulic acid	Phenolic acid		23.96	**[16]**
4	1.20	194		193	178, 161, 137, 134, 133	Isoferulic acid	Phenolic acid	20.82		**[57]**
5	1.41	272	273	271	253, 161, 135, 134, 91	Butin isomer (Butein)	Chalcone	23.01		**[55,56]**
6	1.96	256		255	213, 135, 119, 91	Liquiritigenin (7,4'-dihydroxy flavanone)	Chalcone	3.89		**[54]**
7	2.17	286	287	285	257, 151, 133, 93	Luteolin	Flavone	1.22		**[53]**
8	2.40	300		299	271, 151, 133, 93	Diosmetin	Flavone	5.38		**[52]**
9	2.40	300		299	299	Grandiflorenic acid	Diterpene		2.62	**[26]**
10	3.15	498		497	318[M^{+}-H-glucose] ,203,185,165	Naueloside G	Glycosides		3.89	**[51]**
11	3.67	286	287		258, 241, 231, 213, 165, 153, 137, 121, 111	Kaempferol	Flavonol	1.05		**[49,50]**

12	4.23	318	319		319[M^++H],274[M^+-COOH]	Hydroxy entkaurenoic acid or grandifloric acid	Diterpene		3.35	**[26]**
13	15.86	280	281		281[M^++H],280[M^++H-H], 263[M^++H-H_2O]	Linolelaidic acid	Fatty acid		3.83	
14	17.36	302		301	301, 286, 283, 165, 150, 135	Hesperetin	Flavanone	6.60		**[46-48]**
15	18.93	282	283		266,239,237,225,195,177,166, 164,133,89	Hexaethylene glycol	Alcohol		26.01	**[45]**
16	19.90	608	609		505(Bp)[M^++H-104(benzoyl moiety)], 203[M^++H-104-302(entkaurenoic acid)],122	entkaurenoic acid derivative	Diterpene		0.82	**[6]**
17	22.45	284	285			Stearic acid	Fatty acid		5.73	
18	36.00	146		145		Coumarin	Coumarin		3.89	**[24]**

LC–MS/MS-only assignments represent tentative candidate structures. Distinct retention times and fragmentation patterns were used to distinguish chromatographic features, but exact positional and stereochemical identities require confirmation using authentic standards or complementary spectroscopic methods.

Supplementary Table S22. Complete SwissADME-predicted ADME and drug-likeness properties of the non-redundant metabolites identified in STF-EAF and STL-EAF.

Compound	Fraction(s)	MW	HBA	HBD	TPSA	Consensus LogP	ESOL class	GI absorption	BBB permeant	P-gp substrate	Lipinski violations	Bioavailability score
3,4-Dicaffeoylquinic acid	STF-EAF	516.45	12	7	211.28	0.67	Soluble	Low	No	Yes	3	0.11
Butin	STF-EAF; STL-EAF	272.25	5	3	86.99	1.72	Soluble	High	No	Yes	0	0.55
8,3′,4′-Trihydroxyflavone	STF-EAF	270.24	5	3	90.90	1.39	Soluble	High	No	No	0	0.55
Butein	STF-EAF	272.25	5	4	97.99	2.03	Soluble	High	No	No	0	0.55
Caffeic acid	STL-EAF	180.16	4	3	77.76	0.92	Very soluble	High	No	No	0	0.56
Naringenin-7-*O*-glucoside	STL-EAF	434.39	10	6	166.14	-0.31	Soluble	Low	No	No	1	0.55
Valine	STL-EAF	117.15	3	2	63.32	-1.24	Highly soluble	High	No	No	0	0.55
Maltonic acid	STL-EAF	196.16	7	6	138.45	-3.01	Highly soluble	Low	No	No	1	0.56
Ferulic acid	STL-EAF	194.18	4	2	66.76	1.29	Soluble	High	Yes	No	0	0.85
4′-Hydroxy-5,6,7-trimethoxyflavone	STL-EAF	328.32	6	1	78.13	2.01	Soluble	High	Yes	No	0	0.55
3,5,8-Trihydroxy-6,7-dimethoxyflavone	STL-EAF	330.29	7	3	109.36	1.49	Soluble	High	No	No	0	0.55
3,3′,4′,5,5′,7-Hexahydroxyflavone	STL-EAF	318.24	8	6	151.59	0.38	Soluble	Low	No	No	1	0.55
Coumarin	STL-EAF	146.14	2	0	30.21	1.20	Very soluble	High	Yes	No	0	0.55
4′,7-Dihydroxyflavanone	STL-EAF	256.25	4	2	66.76	2.15	Soluble	High	Yes	Yes	0	0.55
7,4′-Dimethoxyflavone	STL-EAF	282.29	4	0	48.67	2.51	Soluble	High	Yes	No	0	0.55

Supplementary Table S23. Detailed interaction parameters of selected metabolite-target complexes visualized in 2D and 3D docking maps.

Fraction	Target (PDB ID)	Ligand	Docking score (kcal/mol)	Interaction type	Interacting residue	Distance (Å)
STF-EAF	STAT3 (6QHD)	3,4-Dicaffeoylquinic acid	−7.3	Conventional hydrogen bond	ASP B:719	2.55 and 2.75
					ARG B:720	4.97
					ASP B:938	2.00
				Carbon hydrogen bond	PRO B:718	2.61
					ASN B:939	2.70
				π-Alkyl	ARG B:720	5.38
		8,3′,4′-Trihydroxyflavone	−7.0	Conventional hydrogen bond	LYS A:417	2.39
				π-Anion	ASP A:414	3.20 and 4.09
				Amide-π stacked	ILE A:413	4.81
				π-Alkyl	PRO A:318	4.38
					LYS A:417	4.73
	EGFR (5U8L)	3,4-Dicaffeoylquinic acid	−9.0	Conventional hydrogen bond	CYS A:74	2.70
					CYS A:96	2.34
					ARG A:140	2.49
				Carbon hydrogen bond	GLY A:18	2.77 and 2.97
				π-π Stacked	PHE A:155	4.84
				π-Alkyl	ALA A:21	5.49
					VAL A:25	4.61
					ALA A:42	4.19
					LEU A:143	4.66
		8,3′,4′-Trihydroxyflavone	−8.0	Conventional hydrogen bond	CYS A:74	2.82
					GLN A:90	2.49 and 2.93
					MET A:92	2.49
					GLY A:95	2.74
				π-Sulfur	CYS A:74	5.81
				π-Sigma	LEU A:17	2.93
				π-Alkyl	LEU A:17	3.88
					VAL A:25	4.87
					ALA A:42	4.12
					LEU A:143	4.55
		Butein	−7.8	Conventional hydrogen bond	LYS A:44	2.54 and 2.78
					MET A:92	2.29
				Carbon hydrogen bond	GLY A:95	2.60

				π-π Stacked	PHE A:155	3.95
				π-Alkyl	LEU A:17	5.09
					VAL A:25	5.43
					LEU A:143	4.50
		Butin	−7.7	Carbon hydrogen bond	GLY A:95	2.50
				π-Alkyl	VAL A:25	4.54 and 5.37
					ALA A:42	4.03
					LEU A:143	4.40
	PTGS2 (5IKR)	8,3′,4′-Trihydroxyflavone	−8.8	Conventional hydrogen bond	TYR A:323	2.37
					SER A:498	2.20
				π-Alkyl	VAL A:317	4.90 and 5.17
					LEU A:320	4.66 and 5.27
					ALA A:495	3.59 and 4.52
		Butein	−7.8	Conventional hydrogen bond	TYR A:323	2.26
					TYR A:353	2.94
					MET A:490	2.02
					SER A:498	2.32
				π-Sulfur	MET A:490	5.98
				π-π T-shaped	TRP A:355	5.32
				π-Alkyl	VAL A:317	4.73
					LEU A:320	5.14
					ALA A:495	4.04
					LEU A:499	4.75
		Butin	−8.5	Conventional hydrogen bond	TYR A:353	2.87
				π-Alkyl	VAL A:317	4.51
					LEU A:320	5.24
					ALA A:495	3.83
					LEU A:499	5.10
STL-EAF	AKT1 (4GV1)	3,5,8-Trihydroxy-6,7-dimethoxyflavone	−8.0	Conventional hydrogen bond	LYS A:37	2.54
					GLU A:92	2.39
				Carbon hydrogen bond	LYS A:16	3.51
				π-Sigma	VAL A:22	2.51
				π-Sulfur	MET A:139	3.70
				π-Alkyl	LEU A:14	5.22
					VAL A:22	5.14 and 5.27
					ALA A:35	3.81
					ALA A:88	4.76

	7,4′-Dimethoxyflavone	−7.6	Carbon hydrogen bond	GLY A:15	2.62
			π-Anion	ASP A:150	3.46
			π-Sigma	VAL A:22	2.72
			π-Sulfur	MET A:139	3.88
			Alkyl	ALA A:35	4.31
				MET A:85	3.74
			π-Alkyl	VAL A:22	3.84
				ALA A:35	4.56
	4′-Hydroxy-5,6,7-trimethoxyflavone	−7.6	Conventional hydrogen bond	LYS A:16	2.88
			π-Sulfur	MET A:139	3.52
			Alkyl	LYS A:37	4.86
			π-Alkyl	VAL A:22	4.38, 4.87 and 5.11
				ALA A:35	4.21
				ALA A:88	5.27
PTGS2 (5IKR)	7,4′-Dimethoxyflavone	−7.9	Conventional hydrogen bond	ARG A:88	2.79
			Amide-π stacked	GLY A:494	4.03
			Alkyl	MET A:81	5.31
				ILE A:313	4.44
				VAL A:317	4.46
				LEU A:327	3.60
				LEU A:352	4.33
				LEU A:499	4.43
			π-Alkyl	VAL A:84	5.34
				VAL A:317	4.98 and 4.98
				LEU A:320	4.70
				LEU A:327	5.15
				PHE A:349	5.02
				TYR A:353	4.08
				TRP A:355	4.95
				ALA A:495	3.50, 4.68 and 4.80
				LEU A:499	4.96

Supplementary Table 24. Grid box parameters and native-ligand redocking results for the selected protein targets.

Target	PDB ID	Docking region	Centers (Å)			Sizes (Å)			Native-ligand redocking score (kcal/mol)	RMSD (Å)
			X	Y	Z	X	Y	Z		
STAT3	6QHD	DNA-binding interface	−43.867	−43.923	3.866	35	35	35	N/A	N/A
EGFR	5U8L	ATP-binding pocket	12.732	−5.978	−32.991	25	25	25	−9.7	1.726
PTGS2	5IKR	Cyclooxygenase channel	38.961	2.353	61.503	30	30	30	−9.1	1.852
AKT1	4GV1	ATP-binding pocket	−20.005	4.344	10.740	25	25	25	−8.8	0.397

N/A, not applicable. Native-ligand redocking was not performed for STAT3 because docking targeted the DNA-binding interface.
